# Characteristics of members of IGT family genes in controlling rice root system architecture and tiller development

**DOI:** 10.3389/fpls.2022.961658

**Published:** 2022-08-26

**Authors:** Jianping Zhao, Lihui Jiang, Hanrui Bai, Yuliang Dai, Kuixiu Li, Saijie Li, Xiaoran Wang, Lixia Wu, Qijing Fu, Yanfen Yang, Qian Dong, Si Yu, Meixian Wang, Haiyan Liu, Ziai Peng, Haiyan Zhu, Xiaoyan Zhang, Xie He, Yan Lei, Yan Liang, Liwei Guo, Hongji Zhang, Decai Yu, Yixiang Liu, Huichuan Huang, Changning Liu, Sheng Peng, Yunlong Du

**Affiliations:** ^1^College of Plant Protection, Yunnan Agricultural University, Kunming, China; ^2^State Key Laboratory for Conservation and Utilization of Bio-Resources in Yunnan, Yunnan Agricultural University, Kunming, China; ^3^Key Laboratory of Agro-Biodiversity and Pest Management of Education Ministry of China, Yunnan Agricultural University, Kunming, China; ^4^Division of Life Sciences and Medicine, College of Life Sciences, University of Science and Technology of China, Hefei, China; ^5^CAS Key Laboratory of Tropical Plant Resources and Sustainable Use, Xishuangbanna Tropical Botanical Garden, Chinese Academy of Sciences, Menglun, China; ^6^Yuguopu District Agricultural Comprehensive Service Center, Mengzi, China

**Keywords:** DRO1, auxin transport, root system architecture, tiller, IGT family genes

## Abstract

Root system architecture (RSA) and tiller are important agronomic traits. However, the mechanisms of the IGT family genes regulate RSA and tiller development in different rice varieties remain unclear. In this study, we demonstrated that 38 rice varieties obtained from Yuanyang Hani’s terraced fields with different RSA and could be classified into six groups based on the ratio of root length and width. We found a positive correlation between RSA (including root width, length, and area) and tiller number in most of rice varieties. Furthermore, the IGT family genes *Deeper Rooting 1* (*DRO1*), *LAZY1*, *TAC1*, and *qSOR1* showed different expression patterns when rice grown under irrigation and drought conditions. Moreover, the *qSOR1* gene had higher levels in the roots and tillers, and accompanied with higher levels of *PIN1b* gene in roots when rice grown under drought environmental condition. *DRO1* gene had two single nucleotide polymorphisms (SNPs) in the exon 3 sequences and showed different expression patterns in the roots and tillers of the 38 rice varieties. Overexpression of *DRO1* with a deletion of exon 5 caused shorter root length, less lateral roots and lower levels of *LAZY1*, *TAC1*, and *qSOR1*. Further protein interaction network, microRNA targeting and co-expression analysis showed that *DRO1* plays a critical role in the root and tiller development associated with auxin transport. These data suggest that the RSA and tiller development are regulated by the IGT family genes in an intricate network way, which is tightly related to rice genetic background in rice adapting to different environmental conditions.

## Introduction

Root system architecture (RSA) plays an important role in plant growth. The spatial arrangement of RSA is generally regulated by the formation and number of taproots, lateral roots adventitious roots and root hairs, and also affected by both genetic and environmental conditions (Smith and De Smet, 2012). The IGT family (named from a conserved “GψL(A/T)IGT” motif) genes, including *Deeper Rooting 1* (*DRO1*), *Tiller Angle Control 1* (*TAC1*), *LAZY1*, and *qSOR1* (*quantitative trait locus for SOIL SURFACE ROOTING 1*) (Guseman et al., 2017; Kitomi et al., 2020), are known to be involved in regulation of the root growth angle (Uga et al., 2013; Guseman et al., 2017; Kitomi et al., 2020; Zhao et al., 2021), tiller angle (Yu et al., 2007), and plant branch (Li et al., 2007; Yoshihara and Iino, 2007). The rice auxin efflux carrier from the PIN-FORMED (PIN) family is one of the most important transporters for auxin polar transport (Adamowski and Friml, 2015). The *DRO1* and *LAZY1* genes control root growth angle (Zhao et al., 2021) and branch angle (Li et al., 2007), respectively, involving in regulating polar auxin transport. The C-terminal sequence of rice DRO1 contains an Ethylene-responsive Amphiphilic Repression (EAR)-like motif (IVLEI), which the EAR motif (L × L × L) found in *LAZY1* (Guseman et al., 2017; Taniguchi et al., 2017). The EAR-like motif is required for RSA regulated by the *AtDRO1* in Arabidopsis (Guseman et al., 2017) and for the subcellular localization of *qSOR1* in rice (Kitomi et al., 2020). microRNAs play an important role in plant growth. Previous reports show that *miRNA156* promotes plant drought tolerance (González-Villagra et al., 2017) and regulates temperature-responsive flowering (Hwan Lee et al., 2012), and *miRNA1846* can be detected in rice root, shoot, endosperm, and embryo (Meijer et al., 2022). However, the differential roles of the IGT family genes associated with auxin and microRNAs in rice RSA and tiller development remain unclear.

The Yuanyang Hani’s terraced fields are located at altitudes of 144–2000 m in the south of Ailao Mountain in Yunnan Province, China. Due to the natural conditions and selection by local people, the Yuanyang Hani’s terraced fields have abundant paddy rice landraces (Cui et al., 2017). A local rice variety, Acuce, has particularly long root length and shows strong drought avoidance (Zhao et al., 2021). However, there are more than thirty rice varieties grown on the Yuanyang Hani, and the mechanism of root and tiller development in these rice varieties adapting to different environmental stresses is unclear. We wanted to investigate whether and how the IGT family genes regulate root and tiller development to promote these rice landraces to adapt to the local terraced fields conditions.

In this study, we found that the 38 rice varieties obtained from Yuanyang Hani’s terraced fields had different RSA, and the RSA had a positive correlation with tiller number in most of rice varieties. Furthermore, the IGT family genes showed different expression patterns in the root and tiller development when rice seedlings grown under irrigation or drought conditions. Importantly, a network of IGT family genes including protein interaction, microRNA targeting and co-expression pattern indicates that the *DRO1* gene plays a critical role in RSA and tiller development, which was associated with auxin transport. These findings provide new insights into the root and tiller development regulated by the IGT family genes to adapt to different environmental stresses.

## Materials and methods

### Plant materials and growth conditions

The following rice germplasms are paddy rice landraces and all obtained from the Yuanyang Hani terraced fields, Yunnan Province, China, including Acuce (*Oryza sativa* cv. indica, Acuce), Qi Xian Gu (Qxg), Da Leng Shui (Dls), Lao Jing Hong Jiao (Ljhj), Ma Wei Gu (Mwg), Ye Bai Gu (Ybg), Man Che Hong Nuo (Mchn), Hei Gu (Heig), Duo Dian (Dd), Nuo Gu (Ng), Meng La Gu (Mlag), Che Bu (Cb), Hua Gu (Huag), Jiu Yue Nuo (Jyn), Ma Zha Nuo (Mzn), Ai Zhe Gu (Azg), Chang Wei Nuo (Cwn), Yun Xiang (Yx), Mao Lai Gu (Mlig), Che Zuo (Cz), Ga Niang Hong Gu (Gnhg), Xiao Gu (Xg), Chuan Bai Gu (Cbg), Shi Yue Bai Gu (Sybg), Gan Di Gu (Gdg), Ban Jiu Gu (Bjg), Meng La Nuo (Mln), Xi Bai Gu (Xbg), Xiao Pi Gu (Xpg), Xiao Hua Gu (Xhg), Si Ma Che (Smc), Hong Jiao Lao Jing (Hjlj), Ma Xian Gu (Mxg), Hua Ke Nuo (Hkn), Gan Tian Nuo (Gtn), Le Che Che Ma (Lccm), Da Pi Gu (Dpg), Xiao Hua Nuo (Xhn), and Ai Jiao Gu (Ajg).

To observe the rice agronomic traits, including the root and tiller phenotypes, rice seeds were germinated in the field soil and grown for 3 weeks. The rice seedlings were then transplanted into plastic basins and grown in experimental fields located in Xiao Guo Xi Village, Caoba Town, Mengzi City, Yunnan Province (103°37′67′′ E, 23°49′39′′ N) with irrigation condition and Yunnan Agricultural University, Kunming, Yunnan Province (102°45′29′′ E, 25°8′26′′ N) with drought condition for 5 months, respectively. We repeated the experiment three times in April 2019, 2020, and 2021.

To detect the expression levels of *DRO1* in roots and tillers of the 38 rice varieties from the Yuanyang Hani terraced fields, rice seeds were germinated in soil and grown for 3 weeks, and then the rice seedlings were transplanted into plastic basins and grown in experimental fields located in in Yunnan Agricultural University with drought condition for 3 months in 2022.

### Rice seedling tissue culture

Rice seeds were surface-sterilized with 70% alcohol for 90 s, followed with treatment with 2% sodium hypochlorite for 14 min, and then washed five times with sterilized water. Seeds were cultured on Murashige-Skoog (MS) medium supplemented without sucrose and phytohormone for 3 days under dark condition, and then grown for 4 days under light condition. The 7-day old rice seedlings were used to observe the phenotypes of primary roots and lateral roots, and detect gene expression levels. The lateral roots were counted from primary root emerging lateral root growth point, and calculated the number of lateral roots per unit primary root length.

### Construction of plasmid and transgenic rice lines

To construct the expression vectors *35S::DRO1*Δ*exon5*, the genomic DNA of *DRO1* with deletion of the fourth intron and the fifth exon was amplified from the *pBWA(V)HII-ProDRO1A::DRO1A* plasmid containing complete *DRO1* sequence (GenBank no. MH939159.1). Gene-specific primers DRO1-FP1 and DRO1-RP1 (Supplementary Table 1) were synthesized for PCR amplification. The PCR reaction mixtures were prepared with 4 μL primer pair DRO1-FP1 and DRO1-RP1, 5 μL 10 × PCR Buffer, 5 μL dNTPs (2 mM), 3 μL Mg^2+^ (25 mM), 1 μL Neo enzymes mix (1 U/μL), and 50 ng of plasmid template, then added ddH_2_O to 50 μL. PCR reactions were performed under the following conditions: denaturation at 94°C for 2 min, followed by 29 cycles of 98°C for 10 s, 58°C for 30 s, and 68°C for 1 min. The PCR products were cloned into *BGV002* vector and transcribed under the 35S promoter, and the recombinant expression vector was transferred into the *Agrobacterium tumefaciens* strain EHA105. Rice Acuce transformation was performed as previously described by the *Agrobacterium*-mediated method (Nishimura et al., 2006) at Biogle Company (Hangzhou Biogle Co., Ltd., Hangzhou, China).

### RNA isolation, cDNA synthesis, gene expression, and sequence analysis

Rice roots derived from tissue culture seedlings and rice seedlings grown under experimental fields with irrigation and drought stresses were pooled together. The total RNA isolation and complementary DNA (cDNA) synthesis followed methods described previously (Zhao et al., 2021). The relative quantitative expression levels of the *DRO1*, *LAZY1*, *TAC1*, *qSOR1*, *PIN1b*, *LEA19*, *WAXY*, *ARF15*, *Os03g0624000*, *CRL1*, *Os06g0311000*, *WOX11*, *SHR1*, *PIN1A*, *PIN1C*, and *PIN2* genes were determined using an ABI QuantStudio 7 Flex Real-Time PCR system (Applied Biosystems, Waltham, MA, United States). The 10 μL reaction mixture was prepared with 5 μL PowerUp SYBR Green Master Mix (Thermo Fisher Scientific, Waltham, MA, United States) containing 0.5 μL cDNA template and 0.8 μL primer pairs DRO1-rFP and DRO1-rRP1, LAZY1-rFP and LAZY1-rRP, TAC1-rFP and TAC1-rRP, qSOR1-rFP and qSOR1-rRP, PIN1b-rFP and PIN1b-rRP, LEA19-rFP and LEA19-rRP, WAXY-rFP and WAXY-rRP, ARF15-rFP and ARF15-rRP, Os03g0624000-rFP and Os03g0624000-rRP, CRL1-rFP and CRL1-rRP, Os06g0311000-rFP and Os06g0311000-rRP, WOX11-rFP and WOX11-rRP, SHR1-rFP and SHR1-rRP, PIN1A-rFP and PIN1A-rRP, PIN1C-rFP and PIN1C-rRP, PIN2-rFP and PIN2-rRP (Supplementary Table 1) for *DRO1*, *LAZY1*, *TAC1*, *qSOR1* and, *PIN1b*, *LEA19*, *WAXY*, *ARF15*, *Os03g0624000*, *CRL1*, *Os06g0311000*, *WOX11*, *SHR1*, *PIN1A*, *PIN1C*, and *PIN2*, respectively. The gene-specific primer pairs DRO1-rFP and DRO1-rRP2, DRO1-rFP and DRO1-rRP3 (Supplementary Table 1) were used to detect the expression levels of endogenous *DRO1* and transgene *DRO1* in transgenic rice lines #1, #4, and #8. To detect gene haplotype of *DRO1* in the 38 rice varieties, the gene-specific primer pair DRO1-FP2 and DRO1-RP2 (Supplementary Table 1) were used to amplify the *DRO1* gene. The Actin gene (GenBank no. AK060893) acted as the internal control, and was amplified using the primer pair Actin-FP and Actin-RP (Supplementary Table 1). Real-time PCR was performed under the following conditions: denaturation at 95°C for 2 min, followed by 40 cycles of 95°C for 45 s, 54–58°C for 30 s, and 72°C for 1 min. Three biological replicates were made. The relative expression level was calculated by using the 2^–ΔΔ*Ct*^ method. The PCR products of *DRO1* gene were checked by using electrophoresis on 2% agarose gels and sequenced (Tsingke Biogle Co., Ltd, Beijing, China) to analysis *DRO1* haplotypes.

### Construction of the network among the IGT family proteins and proteins involved in auxin response and root development and microRNAs

The Search Tool for Retrieval of Interacting Genes/Proteins (STRING) database was used to build the protein–protein interaction (PPI) network between the IGT family proteins and proteins related to root development and auxin response, which provides directly physical connections as well as indirectly functional relationships (Szklarczyk et al., 2021). The protein sequences of qSOR1, DRO1, TAC1, and LAZY1 were used as query sequences, and we used STRING to find proteins that interact with them. The co-expression data were downloaded from Rice RNA-seq Database and Rice FREND database (Sato et al., 2013; Yu et al., 2022), which provide rice expression profiles and RNA-seq data. The rice microRNA (miRNA) family mature sequences were retrieved from Plant miRNA Encyclopedia (Guo et al., 2020), which is a database of miRNA loci (MIR) in many species including *O. sativa*. The IGT and PIN family genes were used as target genes for miRNA target prediction study with the psRNATarget program (Dai et al., 2018). Plant miRNA Encyclopedia and psRNATarget are two common tools which are wildly used in plant microRNA target analysis. Next, the PPI network, microRNA regulation network and co-expression network were visualized by Cytoscape (version 3.7.1) (Shannon et al., 2003). Then we combined the PPI network, microRNA regulation network and co-expression network with purple lines, green lines and orange dot lines and highlighted the proteins involved in auxin response and root development with red nodes and green nodes, respectively.

### Data and statistical analysis

Image J 1.41 was used to measure root length, width, growth angle, and surface area. Measurement of the root growth angle followed methods described in previous report (Zhao et al., 2021). SPSS version 19.0 (IBM Inc., Armonk, NY, United States) was used to analyze differences in gene expression and normality distributions of length-width ratios in the 38 rice varieties. *P* < 0.05 indicated a statistical difference and *P* < 0.01 indicated a statistically significant difference. Pearson’s correlation coefficient was calculated by using the SPSS software and was used to analyze correlations between the tiller number and root area, root width, root length, and root length and growth angle. All images were processed in Photoshop.

## Results

### Rice varieties showed different root system architectures

Rice varieties that obtained from Yuanyang Hani’s terraced fields grown under irrigation condition showed different plant height and heading date (Supplementary Figure 1). We further observed the root phenotypes of 38 rice varieties (Supplementary Figure 2). The 38 varieties showed differences in tiller number and root area (Supplementary Table 2), with positive correlations between tiller number and root area, and between tiller number and root width (Supplementary Figure 3). The RSA of these 38 rice varieties could be classified into six groups according to the normality distributions of the ratio of rice root length and width (Supplementary Figure 4). The ratio distributions were 1.3–1.4, 1.4–1.5, 1.5–1.6, 1.6–1.7, 1.7–1.8, and 1.8–1.9, and the architectures of these groups were typified by the races Da Leng Shui (Dls), Hei Gu (Heig), Ai Zhe Gu (Azg), Ban Jiu Gu (Bjg), Ma Xian Gu (Mxg), and Xiao Hua Nuo (Xhn), respectively (Supplementary Figure 2; Supplementary Table 2).

**FIGURE 1:**
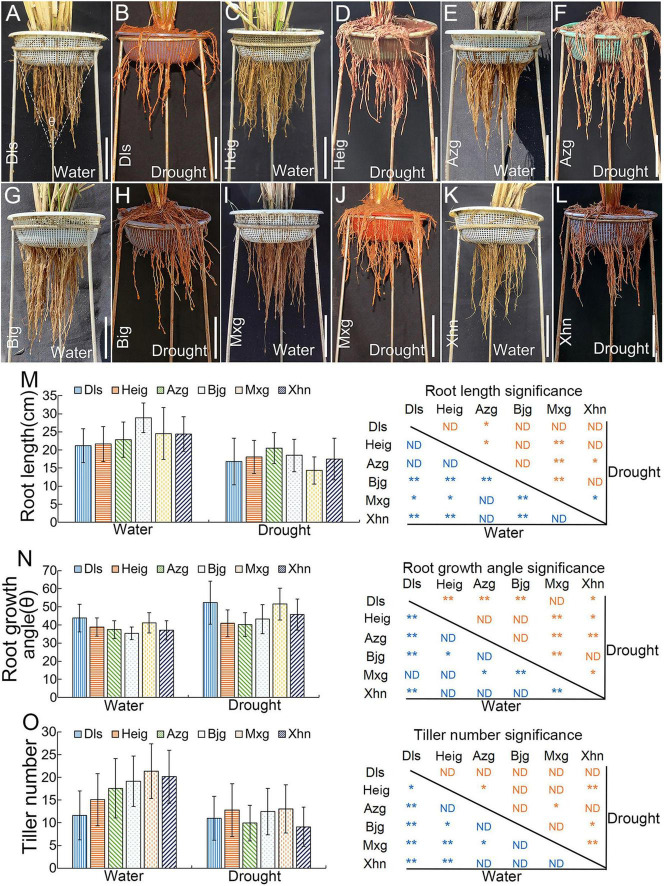
Root and tiller phenotypes of six rice varieties grown under field condition. Six rice varieties Dls **(A,B)**, Heig **(C,D)**, Azg **(E,F)**, Bjg **(G,H)**, Mxg **(I,J)**, and Xhn **(K,L)** were grown under irrigation conditions **(A,C,E,G,I,K)** or drought stress **(B,D,F,H,J,L)** for 5 months. Quantification of root lengths (left) and significance (right) **(M)**, root growth angles (left) and significance (right) **(N)**, tiller numbers (left) and significance (right) **(O)**. Data are means ± SD. *P < 0.05, **P < 0.01 (Tukey’s HSD test). ND, no difference; Dls, Da Leng Shui; Heig, Hei Gu; Azg, Ai Zhe Gu; Bjg, Ban Jiu Gu; Mxg, Ma Xian Gu; Xhn, Xiao Hua Nuo; θ, root growth angle. Scale bar = 10 cm.

To further examined the rice agronomic traits, the six typical rice varieties Dls, Heig, Azg, Bjg, Mxg, and Xhn were grown in experimental fields with irrigation or drought conditions (Figures 1A–L). Compared with the rice varieties grown under irrigation condition (Figures 1A,C,E,G,I,K), rice seedlings showed short root phenotypes when rice grown under drought condition (Figures 1B,D,F,H,J,L). We also found significant differences in root length (Figure 1M), root growth angle (Figure 1N), and tiller number (Figure 1O) between the six rice varieties. In the six rice varieties, compared with rice seedling grown under irrigation condition, the rice varieties Heig and Azg also had small root growth angles when rice seedlings grown under drought stress (Figure 1N), it showed that rice varieties Heig and Azg had deep-rooting phenotype to adapt to drought stress.

**FIGURE 2:**
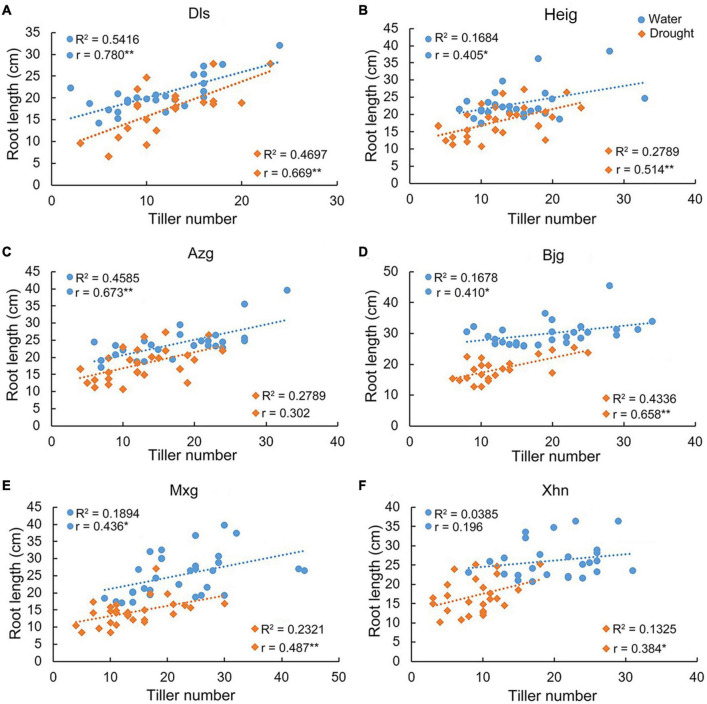
Correlations between tiller numbers and root lengths in different rice varieties. The correlation between root lengths and tiller numbers in six rice varieties shown in Figures 1A–L were analyzed by using SPSS software **(A–F)**. R^2^, determination coefficient; r, Pearson’s correlation coefficient; *r* > 0, positive correlation; *P < 0.05, **P < 0.01 (SPSS analysis). Water, rice seedlings grown under irrigation condition; Drought, rice seedlings grown under drought condition; Dls, Da Leng Shui; Heig, Hei Gu; Azg, Ai Zhe Gu; Bjg, Ban Jiu Gu; Mxg, Ma Xian Gu; Xhn, Xiao Hua Nuo.

**FIGURE 3:**
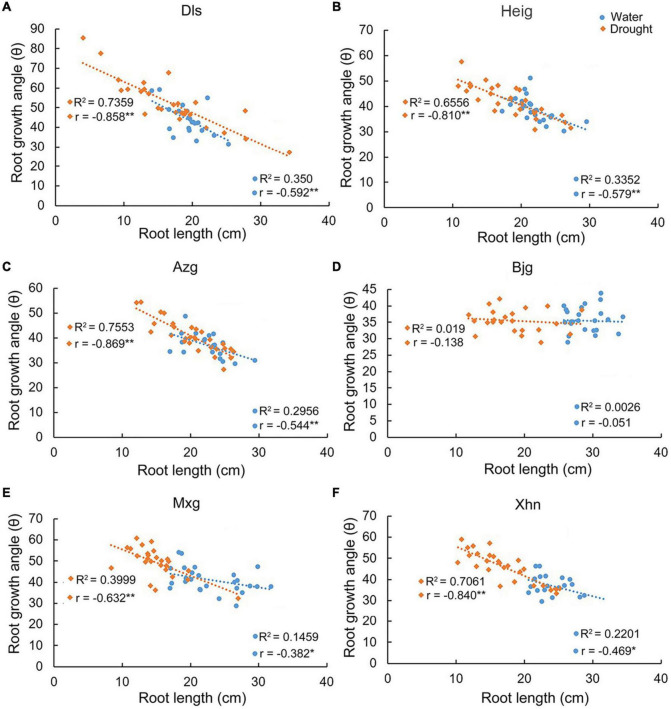
Correlations between root growth angles and root lengths in different rice varieties. The correlation between root lengths and root growth angles in six rice varieties shown in Figures 1A–L were analyzed by using SPSS software. R^2^, determination coefficient; r, Pearson’s correlation coefficient; *r* < 0, negative correlation; *P < 0.05, **P < 0.01 (SPSS analysis) **(A–F)**. Water, rice seedlings grown under irrigation condition; Drought, rice seedlings grown under drought condition; Dls, Da Leng Shui; Heig, Hei Gu; Azg, Ai Zhe Gu; Bjg, Ban Jiu Gu; Mxg, Ma Xian Gu; Xhn, Xiao Hua Nuo.

We found that there were positive correlations between tiller number and root length in the varieties Dls, Heig, Azg, Bjg, and Mxg, but there was no obvious positive correlation in the variety Xhn grown under irrigation condition (Figure 2). However, there were positive correlations between tiller number and root length in the varieties Dls, Heig, Bjg, Mxg, and Xhn, but there was no obvious positive correlation in the variety Azg grown under the drought condition (Figure 2). We analyzed the correlation between root growth angle and root length when rice seedlings grown on irrigation or drought conditions (Figure 3), it showed a significant negative correlation between these two traits in the varieties Dls, Heig, Azg, Mxg, and Xhn, but no significant negative correlation was observed in rice Bjg (Figure 3).

### Rice root and tiller development is regulated by the IGT family genes

**FIGURE 4:**
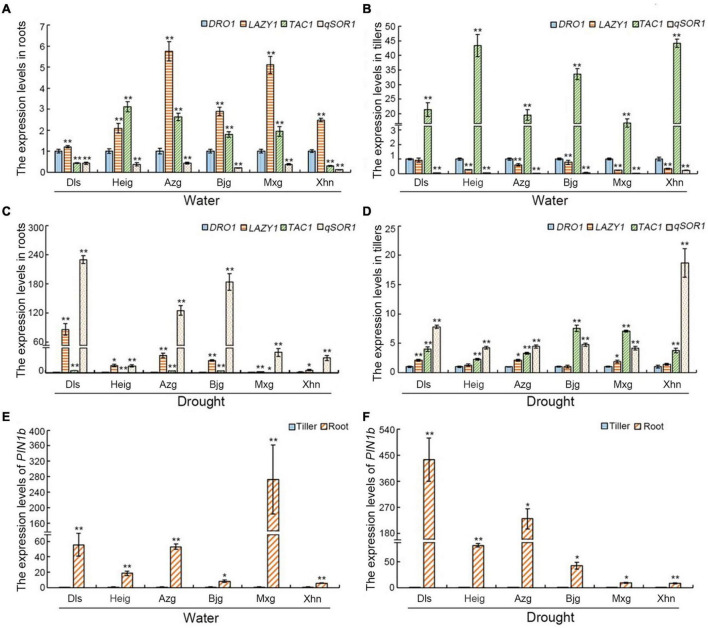
Expression levels of the IGT family genes and *PIN1b* in rice roots and tillers. Five-month-old rice plants grown in the field with irrigation **(A,B,E)** or drought conditions **(C,D,F)**, the expression levels of the IGT family genes *DRO1*, *LAZY1*, *TAC1*, and *qSOR1* in rice roots **(A,C)** and tillers **(B,D)**, and the levels of *PIN1b* gene in rice roots and tillers **(E,F)** were determined by using real-time PCR. The *Actin* gene was used as an internal control. Data are means ± SD. *P < 0.05, **P < 0.01 (SPSS analysis). Dls, Da Leng Shui; Heig, Hei Gu; Azg, Ai Zhe Gu; Bjg, Ban Jiu Gu; Mxg, Ma Xian Gu; Xhn, Xiao Hua Nuo.

To investigate the role of the IGT family genes in rice root and tiller development, we analyzed the expression levels of the *DRO1*, *LAZY1*, *TAC1*, and *qSOR1* genes in these organs. Under irrigation condition, in all varieties studied, compared with the level of *DRO1*, expression levels of *LAZY1* were increased in roots. Furthermore, with the exception of Dls, the expression level of *qSOR1* gene in roots was the lowest of all the genes studied. The expression of these four genes showed a similar pattern (*LAZY1* > *TAC1* > *DRO1* > *qSOR1*) in the varieties Azg, Bjg, and Mxg. The pattern of gene expression (*LAZY1* > *DRO1* > *TAC1* > /≈*qSOR1*) was also similar in the landraces Xhn and Dls, with expression of *TAC1* and *qSOR1* in Dls being almost the same. The variety Heig showed a different gene expression pattern (*TAC1* > *LAZY1* > *DRO1* > *qSOR1*) to the other landraces (Figure 4A). Under drought condition, in all varieties studied, compared with the level of *DRO1*, expression levels of *LAZY1* and *qSOR1* were significantly increased in roots. Furthermore, with the exception of Xhn, the level of *TAC1* expression also was increased in roots. The expression of these four genes showed a similar pattern (*qSOR1* > *LAZY1* > *TAC1* > *DRO1*) in the varieties Dls, Heig, Azg, Bjg, and Mxg. The variety Xhn showed a different gene expression pattern (*qSOR1* > *LAZY1* > *DRO1* > *TAC1*) to the other landraces (Figure 4C).

When we examined the expression levels of the IGT family genes in rice tillers, all rice varieties examined showed similar patterns of gene expression (*TAC1* > *DRO1* > *LAZY1* > *qSOR1*) grown under irrigation condition, although in the case of Dls, the expression levels of *DRO1* and *LAZY1* were very similar. In all varieties, the expression of *TAC1* was very much higher than that of the other examined genes, and that of *qSOR1* was very low (Figure 4B). Furthermore, under drought condition, rice varieties Dls, Heig, Azg, and Xhn showed similar patterns of gene expression (*qSOR1* > *TAC1* > *LAZY1* > *DRO1*), the variety Bjg and Mxg showed a different gene expression pattern (*TAC1* > *qSOR1* > *LAZY1* > *DRO1*) to the other landraces (Figure 4D), although in the rice varieties Heig, Bjg, and Xhn, the expression levels of *DRO1* and *LAZY1* were very similar. Taken together, these results suggest that the *DRO1*, *LAZY1*, *TAC1*, and *qSOR1* genes have different roles in the development of the rice roots and that of the tillers under different growth conditions.

Regulation of auxin distribution by PIN transporters is important in formation of the RSA (Lavenus et al., 2016; Zhao et al., 2021). Therefore, we analyzed the expression levels of *PIN1b* in the rice roots and tillers. When rice varieties were grown on irrigation or drought conditions, we found that the expression levels of *PIN1b* in the roots were significantly higher than that in the tillers (Figures 4E,F), and the increased levels of *PIN1b* in the roots of different rice varieties showed different expression patterns when rice seedlings grown under irrigation condition (Mxg > Dls > Azg > Heig > Bjg > Xhn) or grown under drought stress (Dls > Azg > Heig > Bjg > Xhn > Mxg) (Figures 4E,F).

### Function of *DRO1* gene regulating root growth involves in a cross talk with other members of the IGT family genes

**FIGURE 5:**
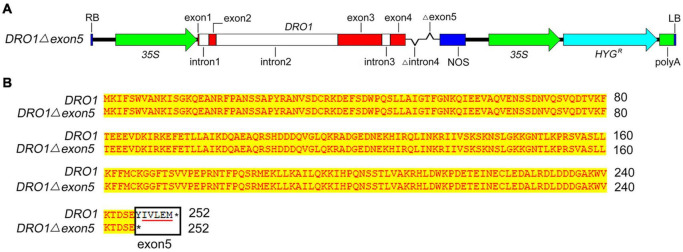
Construction of expression vector containing the *DRO1* gene. Schematic diagram of the expression vector containing *DRO1* deleted the fourth intron and the fifth exon **(A)**. The alignment of amino acid sequences of native *DRO1* and the *DRO1* containing a deletion of the fifth exon sequence expressing in transgenic Acuce rice lines **(B)**. The red boxes indicate exons and white boxes are introns. The black rectangle indicates the fifth exon of *DRO1*, the red line indicates the EAR-like motif in the *DRO1* sequences. 35S, 35S promoter; NOS, nopaline synthase terminator; HYG, hygromycin resistance gene; polyA, polyadenylate; asterisk, protein translation termination.

**FIGURE 6:**
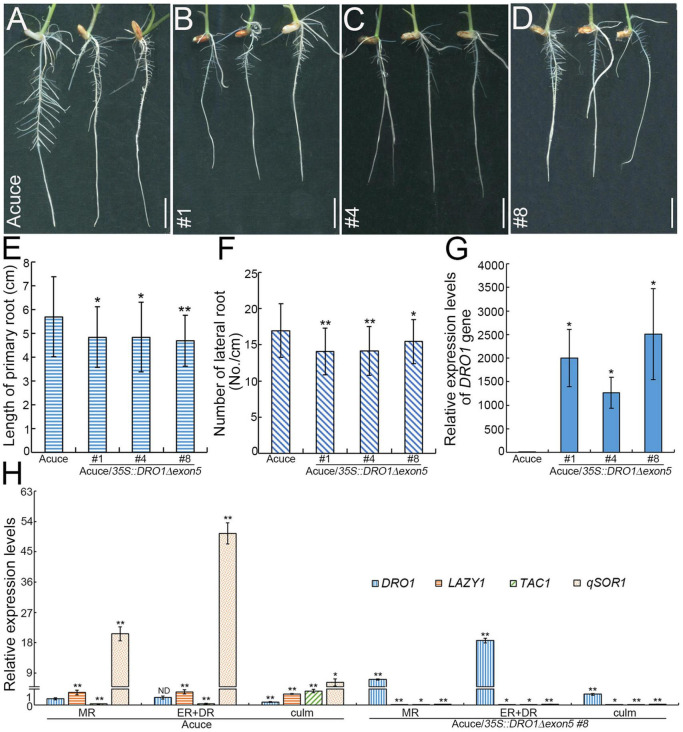
Root phenotypes of transgenic Acuce rice lines. Root phenotypes of wild-type Acuce **(A)** and transgenic rice lines (#1, #4, and #8) overexpressing *DRO1* with a deletion of the fifth exon **(B–D)** grown on 1/2 MS medium. Quantification of the primary root lengths **(E)** (*n*_*Acuce*_ = 43, *n*_*Acuce/*_*_35*S*:*DRO*1_*_Δ_ *_*exon*5 #1_* = 33, *n*_*Acuce/*_*_35*S*:*DRO*1_*_Δ_ *_*exon*5 #4_* = 35, *n*_*Acuce/*_*_35*S*:*DRO*1_*_Δ_ *_*exon*5 #8_* = 39), lateral roots **(F)** (*n*_*Acuce*_ = 57, *n*_*Acuce/*_*_35*S*:*DRO*1_*_Δ_ *_*exon*5 #1_* = 53, *n*_*Acuce/*_*_35*S*:*DRO*1_*_Δ_ *_*exon*5 #4_* = 56, *n*_*Acuce/*_*_35*S*:*DRO*1_*_Δ_ *_*exon*5 #8_* = 40) and the expression levels of the *DRO1* in rice primary roots **(G)**. The levels of the *DRO1*, *LAZY1*, *TAC1*, and *qSOR1* in the primary root and culm of wild-type and transgenic rice line #8 were determined by real-time PCR **(H)**. The *Actin* gene was used as an internal control. Data are means ± SD. *P < 0.05, **P < 0.01 (Student’s *t*-test for root length analysis, SPSS analysis for gene expression). MR, meristem region; ER, elongation region; DR, differentiation region; ND, no difference. Bar = 1 cm.

To further detect the role of *DRO1* in regulating rice root growth, we analyzed the *DRO1* expression patterns of rice roots and tillers in the 38 rice varieties grown under drought condition. Compared with levels of *DRO1* in tillers, 24 rice varieties had higher *DRO1* levels, 12 rice varieties showed lower levels, and two rice varieties had no obvious differences in roots (Supplementary Figure 5). When we analyzed the *DRO1* gene haplotype, it showed two single nucleotide polymorphisms (SNPs) (C-T, A-C) located in the exon 3 in the 38 rice varieties (Supplementary Figure 6). Next, we constructed transgenic Acuce rice lines overexpressing *DRO1* gene with a deletion of the fifth exon (*35S::DRO1*Δ*exon5*) that contains an EAR-like motif (Figures 5A,B). We wanted to test whether the EAR-like motif could be responsible for the role of *DRO1* in regulating rice RSA. It showed that the T2 homozygous transgenic rice lines showed shorter root length (Figures 6B–E; Supplementary Figures 7B–E), less lateral roots (Figure 6F), and had no obvious differences but with decreased tendency in the tiller number (Supplementary Figure 7F) when compared with the wild type (Figure 6A; Supplementary Figure 7A). We found that there were higher levels of *DRO1* in the primary roots in the transgenic rice lines (#1, #4, and #8) than the wild type (Figure 6G). When we detected the transgene *DRO1* levels of primary root in the transgenic rice lines (#1, #4, and #8), the levels of transgene *DRO1* were higher than the endogenous *DRO1* gene (Supplementary Figure 8). This result suggests that the EAR-like motif located in the fifth exon is required for the *DRO1* in regulating rice RSA.

To explore the effect of *DRO1* on other the IGT family members, we tested the expression levels of *DRO1*, *LAZY1*, *TAC1*, and *qSOR1* in the primary root of wild type and transgenic rice lines. It showed that there was a similar expression pattern (*qSOR1* > *LAZY1* > *DRO1* > *TAC1*) in the meristem region, the elongation and differentiation zones in wild-type Acuce seedlings, and there was a different expression pattern (*qSOR1* > *TAC1* > *LAZY1* > *DRO1*) in the culm of wild-type Acuce seedlings (Figure 6H). However, we found that the expression levels of *TAC1*, *LAZY1*, and *qSOR1* were decreased in the meristem region, the elongation and differentiation zones of primary root and culm in the transgenic rice lines (Figure 6H). These data indicate that the malfunction of *DRO1*Δ*exon5* affects the expression of *TAC1*, *LAZY1*, and *qSOR1* genes.

### Network analysis of the IGT family genes

**FIGURE 7:**
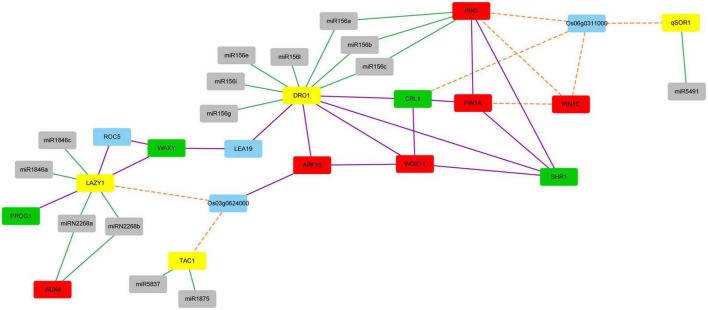
The network among proteins from the IGT family, proteins involved in auxin response and root development and microRNAs. Interacting proteins were connected by purple lines. Proteins with co-expression patterns were linked by orange dot lines. microRNAs and its target genes were linked by green lines. The IGT family proteins were represented by yellow nodes. Proteins involved in auxin response were represented by red nodes, while proteins involved in root development were represented by green nodes, microRNAs were represented by gray nodes, other proteins were represented by blue nodes.

To find the relationship between the IGT family genes and other genes involved in auxin response and root development, a PPI network combined with co-expression network and microRNA targeting network was constructed. A total of 17 proteins and 14 microRNAs were showed in the network (Figure 7). Figure 7 showed that five proteins directly interacted with DRO1 protein, while three proteins directly interacted with LAZY1 protein. It also showed that genes *qSOR1*, *TAC1*, *LAZY1*, and *DRO1* were regulated by different number of microRNAs.

**FIGURE 8:**
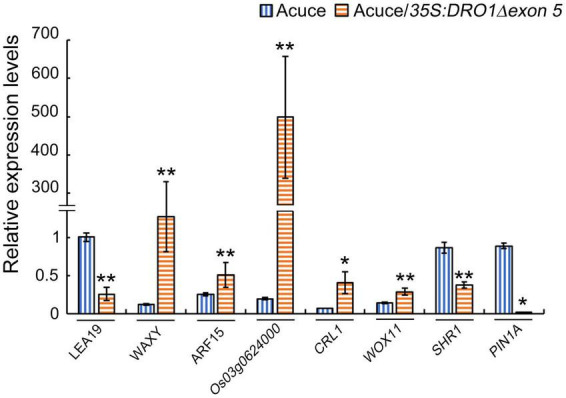
Expression levels of *DRO1* interacting proteins in transgenic rice line. Relative expression levels of *LEA19*, *WAXY*, *ARF15*, *Os03g0624000*, *CRL1*, *WOX11*, *SHR1*, and *PIN1A* genes in the primary roots of rice seedlings Acuce and transgenic rice line #8 were detected by real-time PCR. The *Actin* gene was used as an internal control. The data presented here represent at least three biological replicates. Data are means ± SD; *P < 0.05, **P < 0.01 (SPSS analysis).

It was reported that protein WOX11 may interacted with protein CRL1 and ARF15 (Zhang et al., 2018), and ARF family genes may regulate *DRO1* gene (Mai et al., 2014). These relationships also showed in our predicted network according to Figure 7. Protein PROG1, which has function in controlling tiller angle and number (Jin et al., 2008), interacted with protein LAZY1. This showed that *LAZY1* might be involved in controlling roots and tillers, which is consistent with previously reported function of this gene (Taniguchi et al., 2017). Protein WOX11 and auxin response factor 15 (*ARF15*), which were involved in auxin response, interacted with DRO1, WOX11 was thought to be an integrator of auxin and cytokinin signaling, and it was involved in regulating the cell proliferation during crown root growth (Zhao et al., 2009). SHR1 and CRL1, which were involved in root development, also interacted with DRO1. SHR1 plays an important role in cell division and tracheary element development of roots (Xing et al., 2021), while the CRL1, which is the target of auxin response factor 1, plays a significant role in crown root formation (Inukai et al., 2005). It is suggested that DRO1 might be involved in the auxin related pathway in addition to the root development. Our network also showed that CRL1 had an indirect interaction with ARF15. What’s more, proteins of LAZY1 and DRO1 may indirectly interact with each other through protein LEA19 and WAXY, and DRO1 had an indirect interaction with PIN family proteins according to the Figure 7. To further detect the regulation of DRO1 and its interaction partners, we tested gene expression levels that directly or indirectly interact with DRO1 showed in Figure 7 in the transgenic rice line (#8). Compared with the wild type, the levels of *LEA19*, *SHR1*, and *PIN1A* were decreased, the levels of *WAXY*, *ARF15*, *Os03g0624000*, *CRL1*, and *WOX11* were significantly increased in the primary roots of transgenic rice line (#8) (Figure 8). Based on this result, we speculated that *DRO1* may regulate the levels of *DRO1* interacting partners to affect the expression of other IGT family genes (Figure 6H) in root and tiller development, which associated with changes in auxin transport.

**FIGURE 9:**
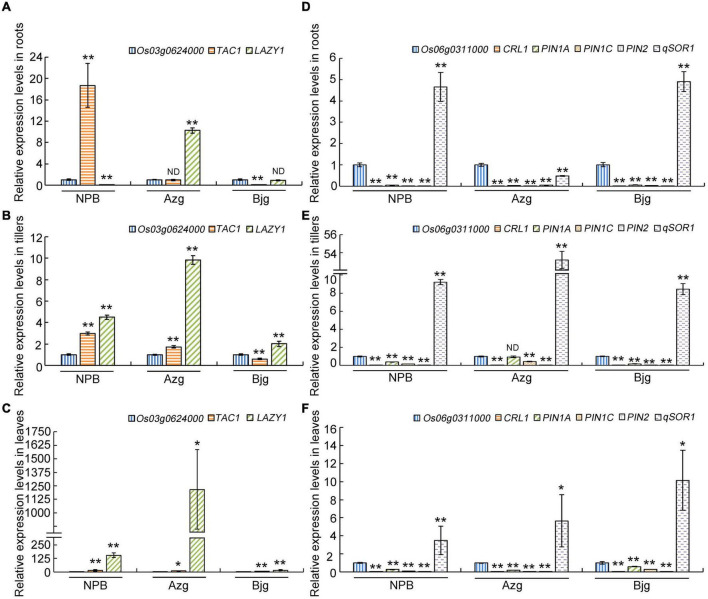
Gene co-expression detection in different rice varieties. Roots, tillers and leaves of 3-month old rice seedlings grown under drought condition were used to detect gene co-expression levels **(A–F)**. The levels of *Os03g0624000*, *LAZY1*, *TAC1* **(A–C)**, *Os06g0311000*, *CRL1*, *PIN1A*, *PIN1C*, *PIN2*, and *qSOR1* **(D–F)** in rice roots **(A,D)**, tillers **(B,E)** and leaves **(C,F)** were determined by using real-time PCR. The *Actin* gene was used as an internal control. The data presented here represent at least three biological replicates. Data are means ± SD. *P < 0.05, **P < 0.01 (SPSS analysis). ND, no difference; NPB, Nipponbare; Azg, Ai Zhe Gu; Bjg, Ban Jiu Gu.

According to Figure 7, *TAC1*, Os03g0624000, and *LAZY1* displayed co-expression patterns, suggesting that *TAC1* and *LAZY1* may be involved in the same biological process. What’s more, *qSOR1*, *PIN* family genes and *CRL1* exhibited co-expression patterns with *Os06g0311000*. We randomly selected rice varieties Azg, Bjg, and Nipponbare (NPB) to detect gene expression patterns, and it showed that the levels of *TAC1*, *Os03g0624000*, and *LAZY1* (Figures 9A–C) and *Os06g0311000*, *CRL1*, *PIN1A*, *PIN1C*, *PIN2*, and *qSOR1* (Figures 9D–F) had similar expression in roots, tillers and leaves, respectively. We found that *PIN1A*, *PIN1C*, *PIN2* had lower expression levels in the roots of rice varieties NPB, Azg, and Bjg (Figure 9D), and *qSOR1* had higher levels in the roots of rice NPB and Bjg, but lower level in rice variety Azg (Figure 9D). PIN family proteins have a critical function in auxin transportation and root development (Li et al., 2019). We hypothesized that *qSOR1* has a role in responding to auxin transport of rice roots based on the network of IGT family genes.

The regulatory relationship between the IGT family genes and mircroRNAs were also displayed in Figure 7. We found that the IGT family genes were regulated by different microRNAs (Supplementary Table 3). It showed that *qSOR1* was only targeted by *miR5491*, while *DRO1* was targeted by seven microRNAs (Supplementary Table 3). The *DRO1* gene was targeted by *miRNA156* family (Supplementary Table 3). *DRO1* and *PIN2* were both target genes of *miR156a*, *miR156b*, and *miR156c*, while *LAZY1* and *AUX4* were targeted by *miRN2268a* and *miRN2268b* (Supplementary Table 3). These microRNAs regulation network suggested that function of the IGT family genes are tightly connected with microRNAs in regulating root and tiller development associated with auxin transport.

## Discussion

Root system architecture is an important agronomic trait in plant adapts to different environmental stresses. In this study, we found that RSA and the expression patterns of the IGT family genes were significantly different in 38 rice varieties from the Yuanyang Hani terraces field. The RSA observed in the 38 rice landraces could be classified into six types. The root area, width and length showed positive correlations with the numbers of tillers, and root growth angle was negatively correlated with root length. The IGT family genes *DRO1*, *TAC1*, *LAZY1*, and *qSOR1* and auxin efflux carrier related gene *PIN1b* showed different expression patterns in the roots and tillers when the rice was grown under irrigation or drought conditions. Importantly, the IGT family genes, auxin response genes, root development genes and microRNA could form a protein interaction and co-expression network, the expression of *DRO1* could affect the levels of *TAC1*, *LAZY1*, and *qSOR1* in the rice root growth.

Altitude is the main factor in determining rice growth in the Yuanyang Hani’s terraced field. Rice varieties, including Lao Jing Hong Jiao, are able to grow at altitudes between 1200 and 1650 m (Cui et al., 2008). In this study, the rice Lao Jing Hong Jiao showed the smallest ratio of root length and width grown under irrigation condition (Supplementary Table 2), rice varieties Dls, Heig, Azg, Bjg, Mxg, and Xhn showed short root phenotypes but rice varieties Heig and Azg had small root growth angle (Figure 1N) when seedlings grown under drought condition (Figure 1), and rice Acuce, which is a dominant variety grown in the Yuanyang Hani’s terraced field, has deeper and longer root phenotypes, and shows strong drought avoidance (Zhao et al., 2021). It indicates that rice varieties adapt to different environmental stresses by using different RSA. We noticed that the *DRO1* gene had higher levels in most of rice varieties grown under drought condition (Supplementary Figure 5), it implies that *DRO1* may play a critical role in controlling RSA development in the IGT family genes.

Genetic background and environmental conditions both contribute to the regulation of root development (Malekpoor Mansoorkhani et al., 2014). Root growth angle and root length are important to plant adapts to drought or salinity stresses (Kato et al., 2006; Uga et al., 2009; Kitomi et al., 2020). Some studies show that drought stress induces the plasticity responses in rice root systems (Meister et al., 2014; Kim et al., 2020; Reeger et al., 2021). In this study, we found that the RSA showed different phenotypes when rice seedlings were grown under normal growth conditions and drought stress (Figure 1), however, rice variety Bjg did not show significant negative correlations between root growth angle and root length (Figure 3). The *DRO1* gene had two SNPs in the exon 3, but the exon 3 sequences of *DRO1* gene in rice Bjg did not shown obvious difference compared with its homologous in other rice varieties (Supplementary Figure 6). It suggests that the formation of RSA is tightly related to rice genetic background as seedlings grown under different environmental stresses.

Function of the IGT family genes involves in regulating plant morphogenesis. *TAC1* and *LAZY1* have been shown to regulate shoot architecture (Yoshihara and Iino, 2007; Yu et al., 2007; Dardick et al., 2013; Taniguchi et al., 2017) and the *qSOR1* is known to control RSA by forming shallow-root phenotype and then adapt to salinity stress (Kitomi et al., 2020). In this study, we found that the IGT family genes *DRO1*, *LAZY1*, *TAC1*, and *qSOR1* showed different expression patterns in rice roots and tillers when rice seedlings were grown under irrigation or drought conditions (Figure 4). It implies that the IGT family genes play differential roles during root and tiller development. Function of *DRO1* gene regulating RSA is tightly connected with auxin distribution (Zhao et al., 2021). Based on the network of the IGT family proteins (Figure 7), the expression patterns of *PIN1b* in rice varieties Dls, Heig, Azg, Bjg, Mxg, and Xhn (Figures 4E,F), and co-expression pattern between *qSOR1* and PIN family genes (Figures 9D–F), we speculated that the IGT family genes could control the rice RSA and tiller development mediated by auxin transport. Previous reports suggest that there is an indirectly negative regulation of *LAZY1* function via *TAC1* for tree branch orientation (Hill and Hollender, 2019; Hollender et al., 2020). From the Figure 7, the PPI among WOX11, CRL1, ARF15, and DRO1 were consistent with previous works (Mai et al., 2014; Zhang et al., 2018), and genes including *CRL1*, *PIN1A*, *PIN1C*, *PIN2*, *qSOR1*, *Os06g0311000*, and *TAC1*, *LAZY1*, and *Os03g0624000* showed similar co-expression patterns in different rice varieties (Figure 9), suggesting the co-expression and protein interaction network among IGT family genes could be reliable. Overexpression of malfunctional *DRO1*Δ*exon5* decreased the levels of *TAC1*, *LAZY1*, and *qSOR1* (Figure 6) might be resulted from a change of the expression levels of *DRO1* interaction partners (Figures 7, 8). These data suggest that *DRO1* has a critical role in regulating RSA and tiller development among the members of the IGT family genes. From the microRNA targeting network, the genes of the IGT family were targeted by different microRNAs (Figure 7; Supplementary Table 3). However, the mechanism of microRNA regulating the IGT family genes to control the RSA and tiller development needs to be further explored.

In summary, rice varieties grown on the Yuanyang Hani’s terraced field have different characteristics of RSA and tillers to allow them to adapt to different environmental conditions. Due to different genetic background of rice varieties, the IGT family genes showed different expression patterns in rice root and tiller development. Furthermore, function of the *DRO1* gene, microRNAs and auxin transport were tightly connected to control RSA and tiller development. Further analysis of the regulatory factors in some rice varieties, including Bjg, will reveal the mechanism behind RSA and tiller development, and will be useful in rice breeding in future.

## Accession numbers

Sequence data for the *DRO1*, *qSOR1*, *LAZY1*, *TAC1*, *PIN1b*, *PROG1*, *ROC5*, *WAXY*, *ARF15*, *LEA19*, *WOX11*, *CRL1*, *SHR1*, *PIN1A*, *PIN2*, *PIN1C*, and *AUX4* genes described in this study can be found in the NCBI database under the following accession numbers: Os09g0439800, Os07g0614400, Os11g0490600, Os09g0529300, Os11g0137000, Os07g0153600, Os02g0674800, Os06g0133000, Os05g0563400, Os05g0542500, Os07g0684900, Os03g0149100, Os07g0586900, Os02g0743400, Os06g0660200, Os06g0232300, and Os10g0147400, respectively.

## Data availability statement

The original contributions presented in this study are included in the article/Supplementary material, further inquiries can be directed to the corresponding authors.

## Author contributions

YDu and SP conceived and designed research. YDu, SP, CL, JZ, LJ, HB, and KL wrote the manuscript. YDa, JZ, LJ, HB, KL, and SL conducted experiments and analyzed data. JZ, XW, LW, QF, YY, LJ, QD, SY, MW, YD, HL, ZP, HaZ, XZ, XH, YLei, YLia, and LG contributed to field cultivation and sample collection. YDu, SP, HoZ, DY, YLiu, HH, and CL provided experimental methods and data analysis. All authors read and approved the manuscript.

## References

[B1] Adamowski M. Friml J. (2015). PIN-dependent auxin transport: Action, regulation, and evolution. *Plant Cell* 27 20–32. 10.1105/tpc.114.134874 25604445 PMC4330589

[B2] Cui B. You Z. Yao M. (2008). Vertical characteristics of the Hani terrace paddyfield ecosystem in Yunnan, China. *Front. Biol. China* 3 351–359. 10.1007/s11515-008-0055-5

[B3] Cui D. Tang C. Li J. Xinxiang A. Koh H. J. (2017). Genetic structure and isolation by altitude in rice landraces of Yunnan, China revealed by nucleotide and microsatellite marker polymorphisms. *PLoS One* 12:e0175731. 10.1371/journal.pone.0175731 28423046 PMC5396909

[B4] Dai X. Zhuang Z. Zhao P. X. (2018). psRNATarget: A plant small RNA target analysis server (2017 release). *Nucleic Acids Res.* 46 W49–W54. 10.1093/nar/gky316 29718424 PMC6030838

[B5] Dardick C. Callahan A. Horn R. Ruiz K. B. Zhebentyayeva T. Hollender C. (2013). PpeTAC1 promotes the horizontal growth of branches in peach trees and is a member of a functionally conserved gene family found in diverse plants species. *Plant J.* 75 618–630. 10.1111/tpj.12234 23663106

[B6] González-Villagra J. Reyes-Diaz M. M. Kurepin L. V. (2017). Evaluating the involvement and interaction of abscisic acid and miRNA156 in the induction of anthocyanin biosynthesis in drought-stressed plants. *Planta* 246 299–312. 10.1007/s00425-017-2711-y 28534253

[B7] Guo Z. Kuang Z. Wang Y. Zhao Y. Tao Y. Cheng C. (2020). PmiREN: A comprehensive encyclopedia of plant miRNAs. *Nucleic Acids Res.* 48 D1114–D1121. 10.1093/nar/gkz894 31602478 PMC6943064

[B8] Guseman J. M. Webb K. Srinivasan C. Dardick C. (2017). DRO1 influences root system architecture in *Arabidopsis* and *Prunus* species. *Plant J.* 89 1093–1105. 10.1111/tpj.13470 28029738

[B9] Hill J. L. Hollender C. A. (2019). Branching out: New insights into the genetic regulation of shoot architecture in trees. *Curr. Opin. Plant Biol.* 47 73–80. 10.1016/j.pbi.2018.09.010 30339931

[B10] Hollender C. A. Hill J. L. Waite J. Dardick C. (2020). Opposing influences of TAC1 and LAZY1 on lateral shoot orientation in *Arabidopsis*. *Sci. Rep.* 10:6051. 10.1038/s41598-020-62962-4 32269265 PMC7142156

[B11] Hwan Lee J. Joon Kim J. Ahn J. H. (2012). Role of SEPALLATA3 (SEP3) as a downstream gene of miR156-SPL3-FT circuitry in ambient temperature-responsive flowering. *Plant Signal. Behav.* 7 1151–1154. 10.4161/psb.21366 22899051 PMC3489649

[B12] Inukai Y. Sakamoto T. Ueguchi-Tanaka M. Shibata Y. Gomi K. Umemura I. (2005). Crown rootless1, which is essential for crown root formation in rice, is a target of an auxin response factor in auxin signaling. *Plant Cell* 17 1387–1396. 10.1105/tpc.105.030981 15829602 PMC1091762

[B13] Jin J. Huang W. Gao J. P. Yang J. Shi M. Zhu M. Z. (2008). Genetic control of rice plant architecture under domestication. *Nat. Genet.* 40 1365–1369. 18820696 10.1038/ng.247

[B14] Kato Y. Abe J. Kamoshita A. Yamagishi J. (2006). Genotypic variation in root growth angle in rice (*Oryza sativa* L.) and its association with deep root development in upland fields with different water regimes. *Plant Soil* 287 117–129.

[B15] Kim Y. Yong S. C. Lee E. Tripathi P. Kim K. H. (2020). Root response to drought stress in rice (*Oryza sativa* L.). *Int. J. Mol. Sci.* 21 1513–1534. 10.3390/ijms21041513 32098434 PMC7073213

[B16] Kitomi Y. Hanzawa E. Kuya N. Inoue H. Uga Y. (2020). Root angle modifications by the DRO1 homolog improve rice yields in saline paddy fields. *Proc. Natl. Acad. Sci. U.S.A.* 117:202005911. 10.1073/pnas.2005911117 32817523 PMC7474696

[B17] Lavenus J. Guyomarc’h S. Laplaze L. (2016). PIN transcriptional regulation shapes root system architecture. *Trends Plant Sci.* 21 175–177. 10.1016/j.tplants.2016.01.011 26809639

[B18] Li P. Wang Y. Qian Q. Fu Z. Wang M. Zeng D. (2007). LAZY1 controls rice shoot gravitropism through regulating polar auxin transport. *Cell Res.* 17 402–410. 10.1038/cr.2007.38 17468779

[B19] Li Y. Zhu J. Wu L. Shao Y. Wu Y. Mao C. (2019). Functional divergence of PIN1 paralogous genes in rice. *Plant Cell Physiol.* 60 2720–2732. 10.1093/pcp/pcz159 31410483

[B20] Mai C. D. Phung N. T. To H. Gonin M. Hoang G. T. Nguyen K. L. (2014). Genes controlling root development in rice. *Rice* 7 1–11. 10.1186/s12284-014-0030-5 26224559 PMC4884052

[B21] Malekpoor Mansoorkhani F. Seymour G. Swarup R. Moeiniyan Bagheri H. Ramsey R. Thompson A. J. (2014). Environmental, developmental, and genetic factors controlling root system architecture. *Biotechnol. Genet. Eng. Rev.* 30 95–112. 10.1080/02648725.2014.995912 25652818

[B22] Meijer A. De Meyer T. Vandepoele K. Kyndt T. (2022). Spatiotemporal expression profile of novel and known small RNAs throughout rice plant development focussing on seed tissues. *BMC Genomics* 23:44. 10.1186/s12864-021-08264-z 35012466 PMC8750796

[B23] Meister R. Rajani M. S. Ruzicka D. Schachtman D. P. (2014). Challenges of modifying root traits in crops for agriculture. *Trends Plant Sci.* 19 779–788. 10.1016/j.tplants.2014.08.005 25239776

[B24] Nishimura A. Aichi I. Matsuoka M. (2006). A protocol for *Agrobacterium*-mediated transformation in rice. *Nat. Protoc.* 1 2796–2802. 10.1038/nprot.2006.469 17406537

[B25] Reeger J. E. Wheatley M. Yang Y. Brown K. M. (2021). Targeted mutation of transcription factor genes alters metaxylem vessel size and number in rice roots. *Plant Direct* 5:e00328. 10.1002/pld3.328 34142002 PMC8204146

[B26] Sato Y. Namiki N. Takehisa H. Kamatsuki K. Minami H. Ikawa H. (2013). RiceFREND: A platform for retrieving coexpressed gene networks in rice. *Nucleic Acids Res.* 41 D1214–D1221. 10.1093/nar/gks1122 23180784 PMC3531108

[B27] Shannon P. Markiel A. Ozier O. Baliga N. S. Wang J. T. Ramage D. (2003). Cytoscape: A software environment for integrated models of biomolecular interaction networks. *Genome Res.* 13 2498–2504. 10.1101/gr.1239303 14597658 PMC403769

[B28] Smith S. De Smet I. (2012). Root system architecture: Insights from *Arabidopsis* and cereal crops. *Philos. Trans. R. Soc. B Biol. Sci.* 367 1441–1452. 10.1098/rstb.2011.0234 22527386 PMC3321685

[B29] Szklarczyk D. Gable A. L. Nastou K. C. Lyon D. Kirsch R. Pyysalo S. (2021). The string database in 2021: Customizable protein–protein networks, and functional characterization of user-uploaded gene/measurement sets. *Nucleic Acids Res.* 49 D605–D612. 10.1093/nar/gkaa1074 33237311 PMC7779004

[B30] Taniguchi M. Furutani M. Nishimura T. Nakamura M. Fushita T. Iijima K. (2017). The *Arabidopsis* LAZY1 family plays a key role in gravity signaling within statocytes and in branch angle control of roots and shoots. *Plant Cell* 29 1984–1999. 10.1105/tpc.16.00575 28765510 PMC5590491

[B31] Uga Y. Ebana K. Abe J. Morita S. Okuno K. Yano M. (2009). Variation in root morphology and anatomy among accessions of cultivated rice (*Oryza sativa* L.) with different genetic backgrounds. *Breed. Sci.* 59 87–93. 10.1270/jsbbs.59.87 26081539

[B32] Uga Y. Sugimoto K. Ogawa S. Rane J. Ishitani M. Hara N. (2013). Control of root system architecture by deeper rooting 1 increases rice yield under drought conditions. *Nat. Genet.* 45 1097–1102. 10.1038/ng.2725 23913002

[B33] Xing Y. Wang N. Zhang T. Zhang Q. Du D. Chen X. (2021). SHORTROOT 1 is critical to cell division and tracheary element development in rice roots. *Plant J.* 105 1179–1191. 10.1111/tpj.15095 33231904

[B34] Yoshihara T. Iino M. (2007). Identification of the gravitropism-related rice gene LAZY1 and elucidation of LAZY1-dependent and-independent gravity signaling pathways. *Plant Cell Physiol.* 48 678–688. 10.1093/pcp/pcm042 17412736

[B35] Yu B. Lin Z. Li H. Li X. Li J. Wang Y. (2007). TAC1, a major quantitative trait locus controlling tiller angle in rice. *Plant J.* 52 891–898. 10.1111/J.1365-313X.2007.03284.X 17908158

[B36] Yu Y. Zhang H. Long Y. Shu Y. Zhai J. (2022). Plant public RNA-seq database: A comprehensive online database for expression analysis of ~ 45 000 plant public RNA-Seq libraries. *Plant Biotechnol. J.* 20 806–808. 10.1111/pbi.13798 35218297 PMC9055819

[B37] Zhang T. Li R. Xing J. Yan L. Wang R. Zhao Y. (2018). The YUCCA-auxin-WOX11 module controls crown root development in rice. *Front. Plant Sci.* 9:523. 10.3389/fpls.2018.00523 29740464 PMC5925970

[B38] Zhao Y. Hu Y. Dai M. Huang L. Zhou D.-X. (2009). The WUSCHEL-related homeobox gene WOX11 is required to activate shoot-borne crown root development in rice. *Plant Cell* 21 736–748. 10.1105/tpc.108.061655 19258439 PMC2671696

[B39] Zhao Y. Wu L. Fu Q. Wang D. Li J. Yao B. (2021). INDITTO2 transposon conveys auxin-mediated DRO1 transcription for rice drought avoidance. *Plant Cell Environ.* 44 1846–1857. 10.1111/pce.14029 33576018

