# Supplementary material for: Characteristics of members of IGT family genes in controlling rice root system architecture and tiller development
Source: Front Plant Sci. 2022 Aug 26;13:961658. doi: 10.3389/fpls.2022.961658 (PMC9487910; doi:10.3389/fpls.2022.961658)
Supplement: Supplementary file 1 [file Data_Sheet_1.docx]

**Supplemental data**

**
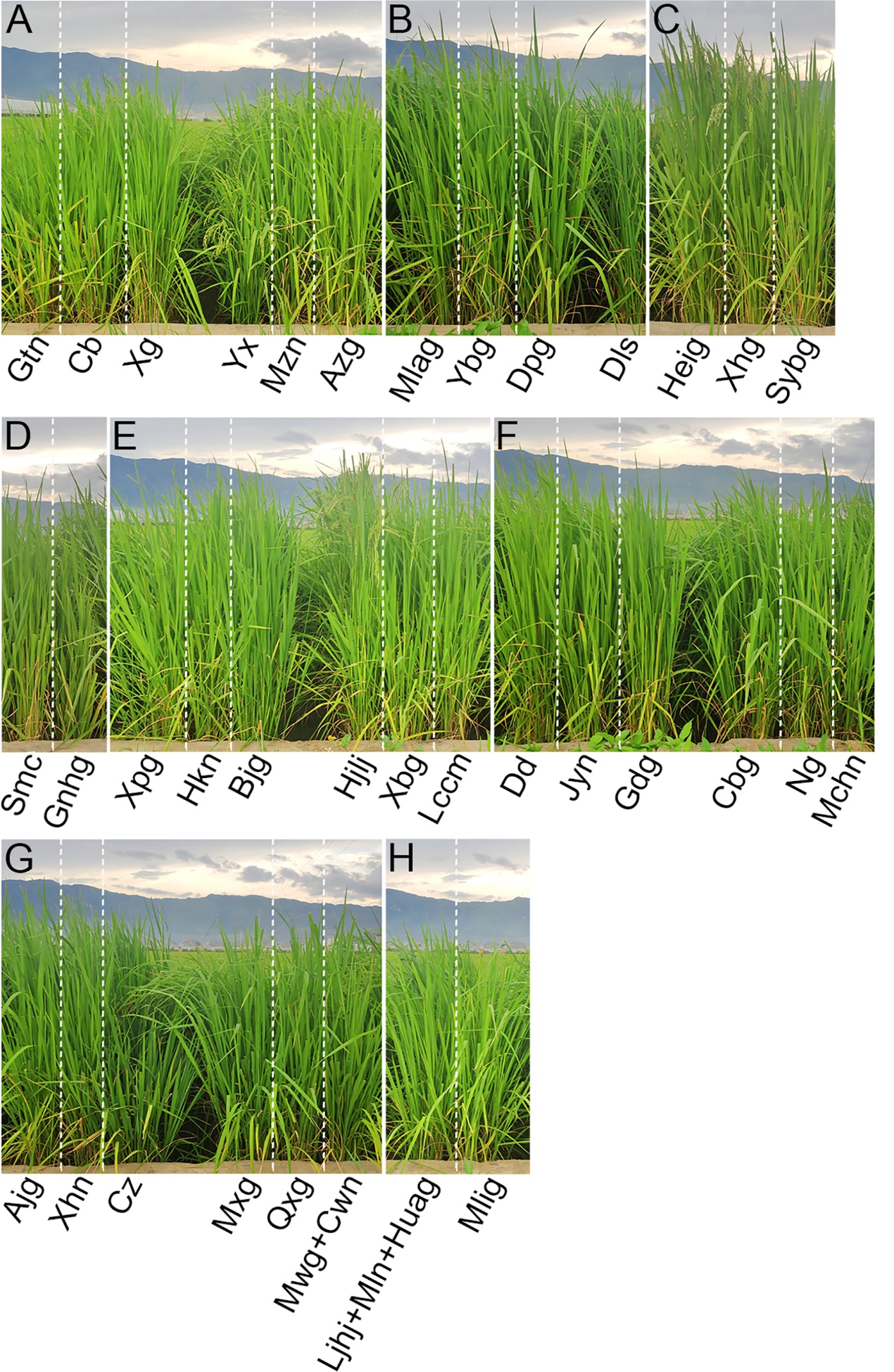
**

Figure S1 Plant phenotypes in the thirty-eight rice varieties grown in field

Rice seedlings Gtn, Cb, Xg, Yx, Mzn, Azg (**A**), Mlag, Ybg, Dpg, Dls (**B**), Heig, Xhg, Sybg (**C**), Smc, Gnhg (**D**), Xpg, Hkn, Bjg, Hjlj, Xbg, Lccm (**E**), Dd, Jyn, Gdg, Cbg, Ng, Mchn (**F**), Ajg, Xhn, Cz, Mxg, Qxg, Mwg, Cwn (**G**), Ljhj, Mln, Huag, Mlig (**H**) were grown in an experimental field with irrigation condition for five months. Gtn = Gan Tian Nuo, Cb = Che Bu, Xg = Xiao Gu, Yx = Yun Xiang, Mzn = Ma Zha Nuo, Azg = Ai Zhe Gu, Mlag = Meng La Gu, Ybg = Ye Bai Gu, Dpg = Da Pi Gu, Dls = Da Leng Shui, Heig = Hei Gu, Xhg = Xiao Hua Gu, Sybg = Shi Yue Bai Gu, Smc = Si Ma Che, Gnhg = Ga Niang Hong Gu, Xpg = Xiao Pi Gu, Hkn = Hua Ke Nuo, Bjg = Ban Jiu Gu, Hjlj = Hong Jiao Lao Jing, Xbg = Xi Bai Gu, Lccm = Le Che Che Ma, Dd = Duo Dian, Jyn = Jiu Yue Nuo, Gdg = Gan Di Gu, Cbg = Chuan Bai Gu, Ng = Nuo Gu, Mchn = Man Che Hong Nuo, Ajg = Ai Jiao Gu, Xhn = Xiao Hua Nuo, Cz = Che Zuo, Mxg = Ma Xian Gu, Qxg = Qi Xian Gu, Mwg = Ma Wei Gu, Cwn = Chang Wei Nuo, Ljhj = Lao Jing Hong Jiao, Mln = Meng La Nuo, Huag = Hua Gu, Mlig= Mao Lai Gu.


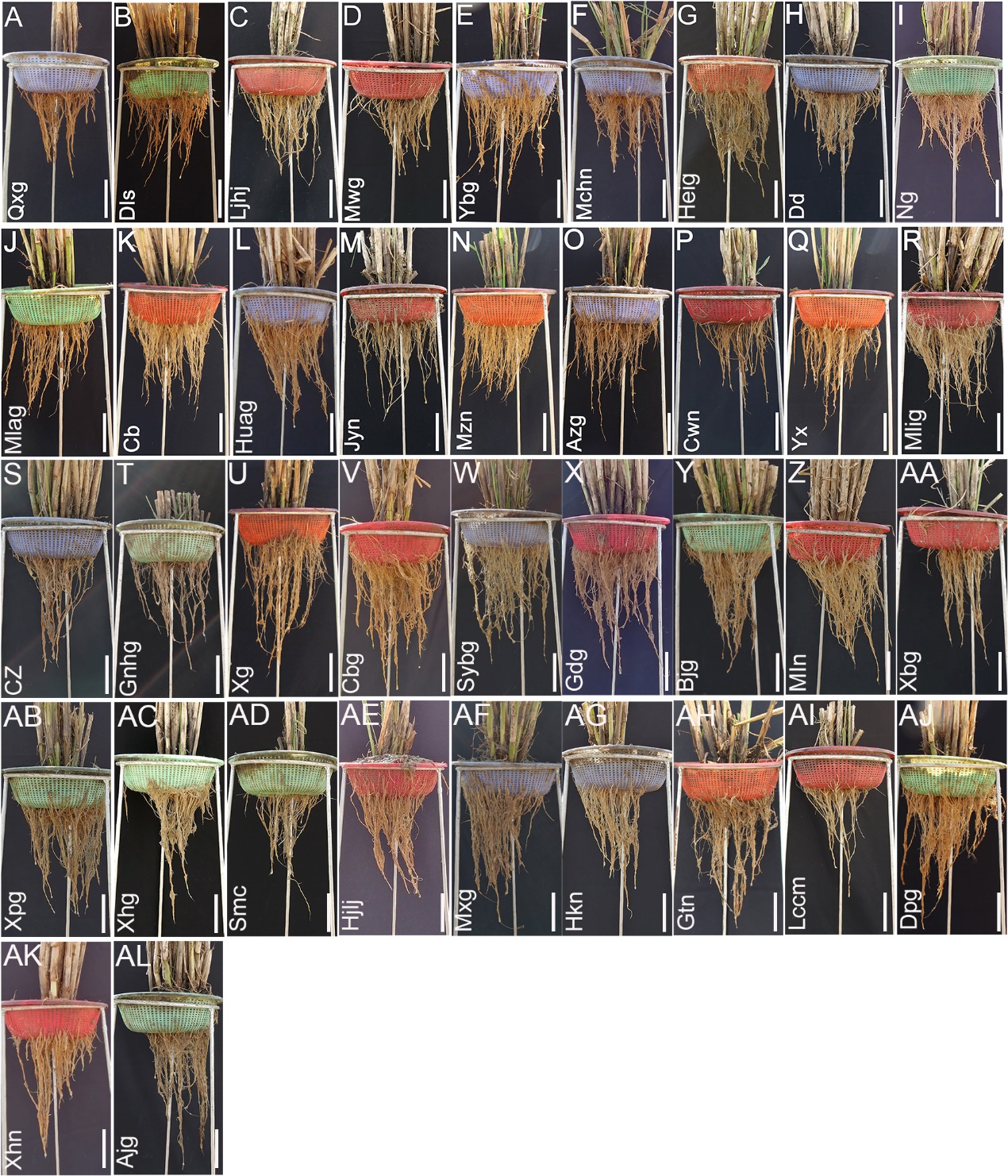


**Figure S2** Root phenotypes of rice varieties grown in the experimental field.

Root phenotypes of five-month-old rice seedlings were grown in an experimental field with irrigation condition. The root length and width were measured, and rice varieties were classified based on the ratio of rice root length and width as follows: ratio 1.3-1.4 (**A-D**), ratio 1.4-1.5 (**E-G**), ratio 1.5-1.6 (**H-R**), ratio 1.6-1.7 (**S-Y**), ratio 1.7-1.8 (**Z-AF**), ratio 1.8-1.9 (**AG-AL**). Qxg = Qi Xian Gu, Dls = Da Leng Shui, Ljhj = Lao Jing Hong Jiao, Mwg = Ma Wei Gu, Ybg = Ye Bai Gu, Mchn = Man Che Hong Nuo, Heig = Hei Gu, Dd = Duo Dian, Ng =Nuo Gu, Mlag = Meng La Gu, Cb = Che Bu, Huag = Hua Gu, Jyn = Jiu Yue Nuo, Mzn = Ma Zha Nuo, Azg = Ai Zhe Gu, Cwn = Chang Wei Nuo, Yx = Yun Xiang, Mlig = Mao Lai Gu, Cz = Che Zuo, Gnhg = Ga Niang Hong Gu, Xg = Xiao Gu, Cbg = Chuan Bai Gu, Sybg = Shi Yue Bai Gu, Gdg = Gan Di Gu, Bjg = Ban Jiu Gu, Mln = Meng La Nuo, Xbg = Xi Bai Gu, Xpg = Xiao Pi Gu, Xhg = Xiao Hua Gu, Smc = Si Ma Che, Hjlj = Hong Jiao Lao Jing, Mxg = Ma Xian Gu, Hkn = Hua Ke Nuo, Gtn = Gan Tian Nuo, Lccm = Le Che Che Ma, Dpg = Da Pi Gu, Xhn = Xiao Hua Nuo, Ajg = Ai Jiao Gu. Scale bar = 10 cm.


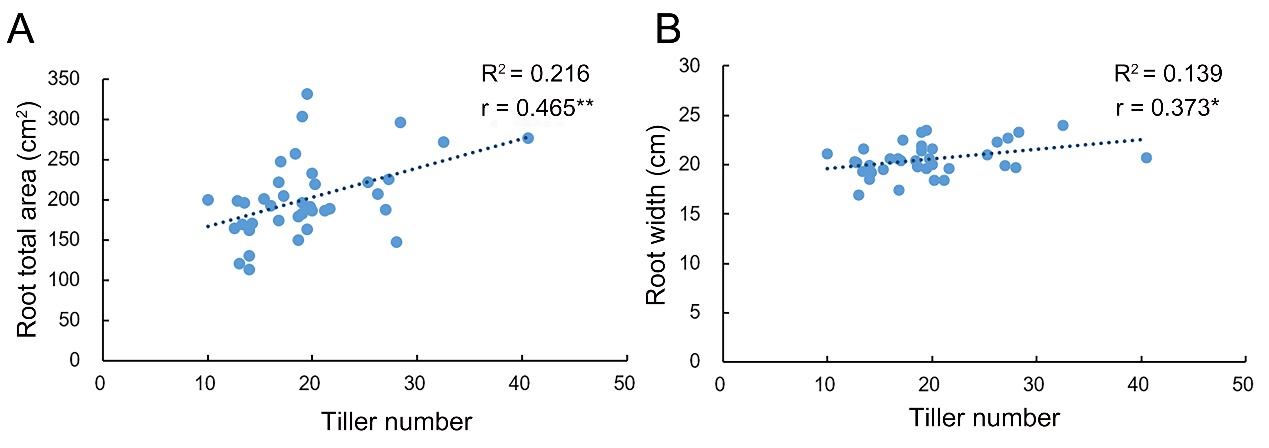


**Figure S3** The correlation of tiller number, total root area and root width.

The correlation between tiller number and root area (**A**), and tiller number and root width (**B**) in the 38 rice varieties shown in **Figures** S**2**. Correlations were analyzed by using SPSS software. R^2^ = determination coefficient, r = Pearson’s correlation coefficient, r > 0, positive correlation, * *P* < 0.05, ** *P* < 0.01 (SPSS analysis).


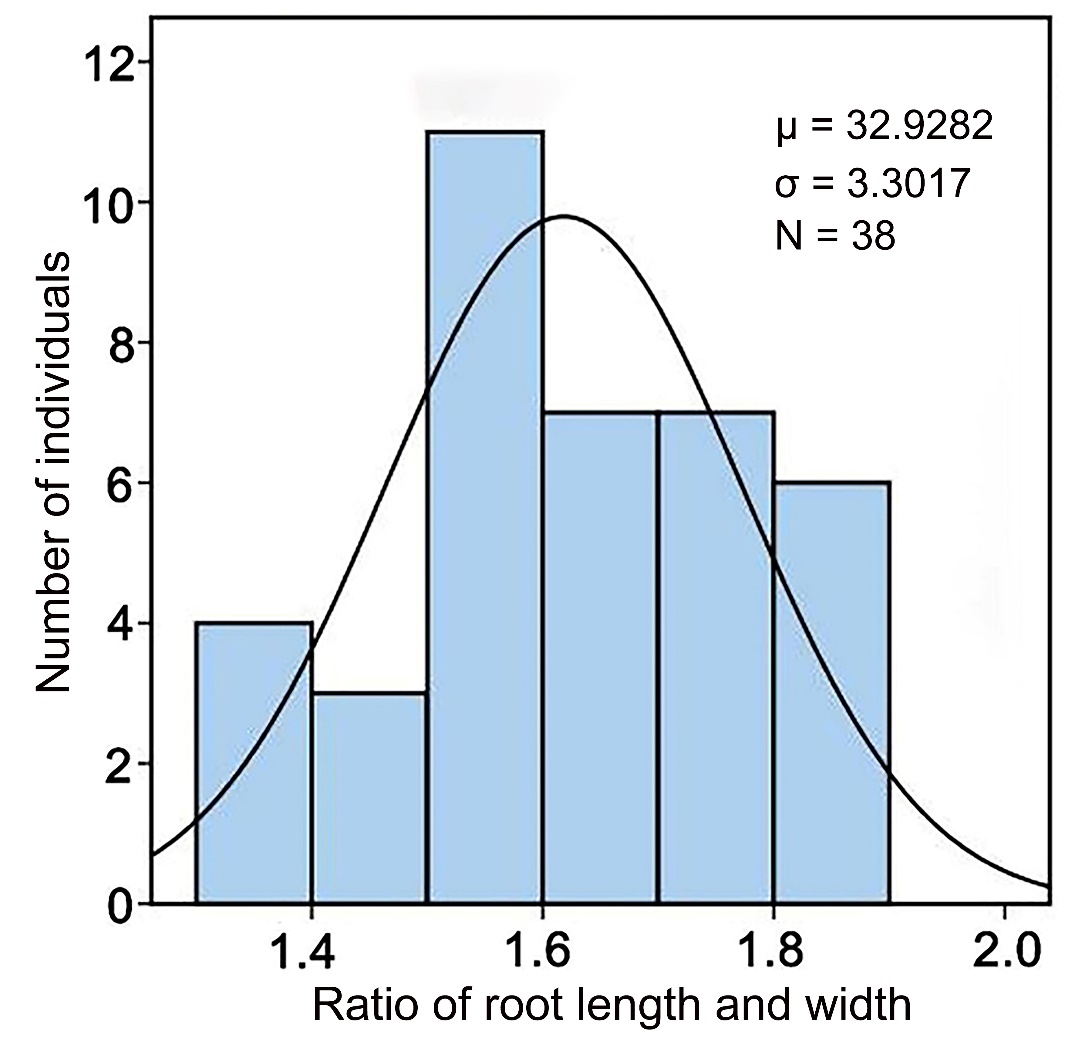


**Figure S4** Histogram showing the ratio of root length and width in the 38 rice varieties.

The root length and width of five-month-old rice seedlings grown in the experimental field were measured. μ = average value, σ = standard deviation, N = sample number.


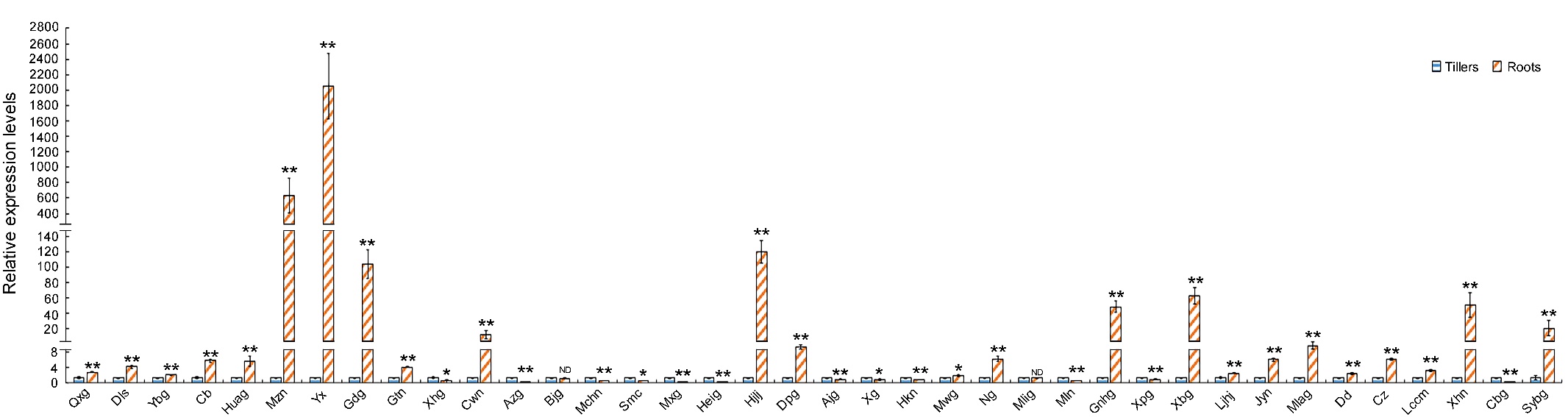


**Figure S5** Expression profile of *DRO1* gene in tillers and roots of thirty-eight rice varieties

Roots and tillers of three-month-old rice seedlings grown under drought condition were used to detect gene expression profile. The expression levels of *DRO1* gene in tillers and roots were detected by real-time PCR. The *Actin* gene was used as an internal control. The data presented here represent at least three biological replicates. Data are means ± SD. * *P* < 0.05, ** *P* < 0.01 (SPSS analysis). ND = no difference. Qxg = Qi Xian Gu, Dls = Da Leng Shui, Ybg = Ye Bai Gu, Cb = Che Bu, Huag = Hua Gu, Mzn = Ma Zha Nuo, Yx = Yun Xiang, Gdg = Gan Di Gu, Gtn = Gan Tian Nuo, Xhg = Xiao Hua Gu, Cwn = Chang Wei Nuo, Azg = Ai Zhe Gu, Bjg = Ban Jiu Gu, Mchn = Man Che Hong Nuo, Smc = Si Ma Che, Mxg = Ma Xian Gu, Heig = Hei Gu, Hjlj = Hong Jiao Lao Jing, Dpg = Da Pi Gu, Ajg = Ai Jiao Gu, Xg = Xiao Gu, Hkn = Hua Ke Nuo, Mwg = Ma Wei Gu, Ng = Nuo Gu, Mlig= Mao Lai Gu, Mln = Meng La Nuo, Gnhg = Ga Niang Hong Gu, Xpg = Xiao Pi Gu, Xbg = Xi Bai Gu, Ljhj = Lao Jing Hong Jiao, Jyn = Jiu Yue Nuo, Mlag = Meng La Gu, Dd = Duo Dian, Cz = Che Zuo, Lccm = Le Che Che Ma, Xhn = Xiao Hua Nuo, Cbg = Chuan Bai Gu, Sybg = Shi Yue Bai Gu.


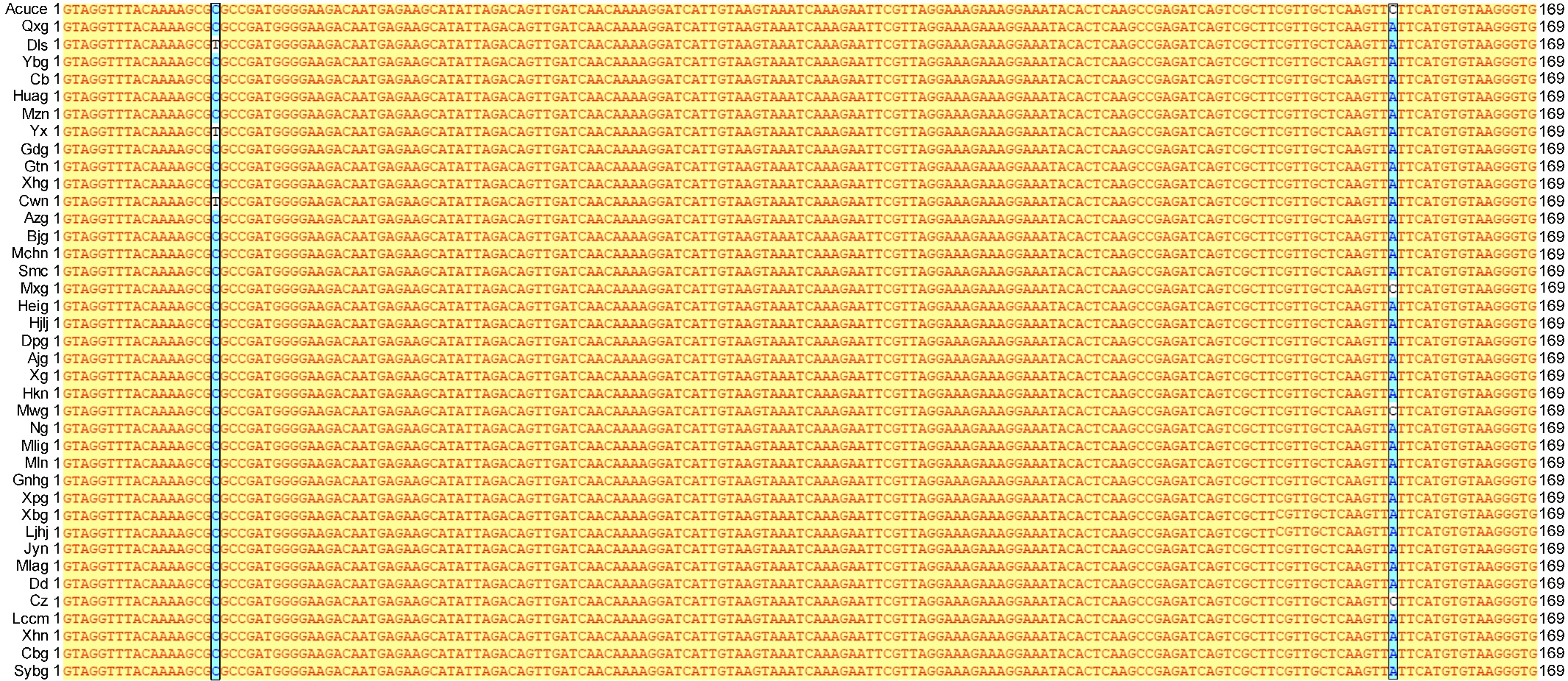


**Figure S6 S**equences alignment of *DRO1* gene in the thirty-eight rice varieties

Partial exon 3 sequences of *DRO1* gene amplified from the roots of the 38 rice varieties were aligned. Black boxes indicate the SNPs in the *DRO1* gene. Qxg = Qi Xian Gu, Dls = Da Leng Shui, Ybg = Ye Bai Gu, Cb = Che Bu, Huag = Hua Gu, Mzn = Ma Zha Nuo, Yx = Yun Xiang, Gdg = Gan Di Gu, Gtn = Gan Tian Nuo, Xhg = Xiao Hua Gu, Cwn = Chang Wei Nuo, Azg = Ai Zhe Gu, Bjg = Ban Jiu Gu, Mchn = Man Che Hong Nuo, Smc = Si Ma Che, Mxg = Ma Xian Gu, Heig = Hei Gu, Hjlj = Hong Jiao Lao Jing, Dpg = Da Pi Gu, Ajg = Ai Jiao Gu, Xg = Xiao Gu, Hkn = Hua Ke Nuo, Mwg = Ma Wei Gu, Ng = Nuo Gu, Mlig= Mao Lai Gu, Mln = Meng La Nuo, Gnhg = Ga Niang Hong Gu, Xpg = Xiao Pi Gu, Xbg = Xi Bai Gu, Ljhj = Lao Jing Hong Jiao, Jyn = Jiu Yue Nuo, Mlag = Meng La Gu, Dd = Duo Dian, Cz = Che Zuo, Lccm = Le Che Che Ma, Xhn = Xiao Hua Nuo, Cbg = Chuan Bai Gu, Sybg = Shi Yue Bai Gu.


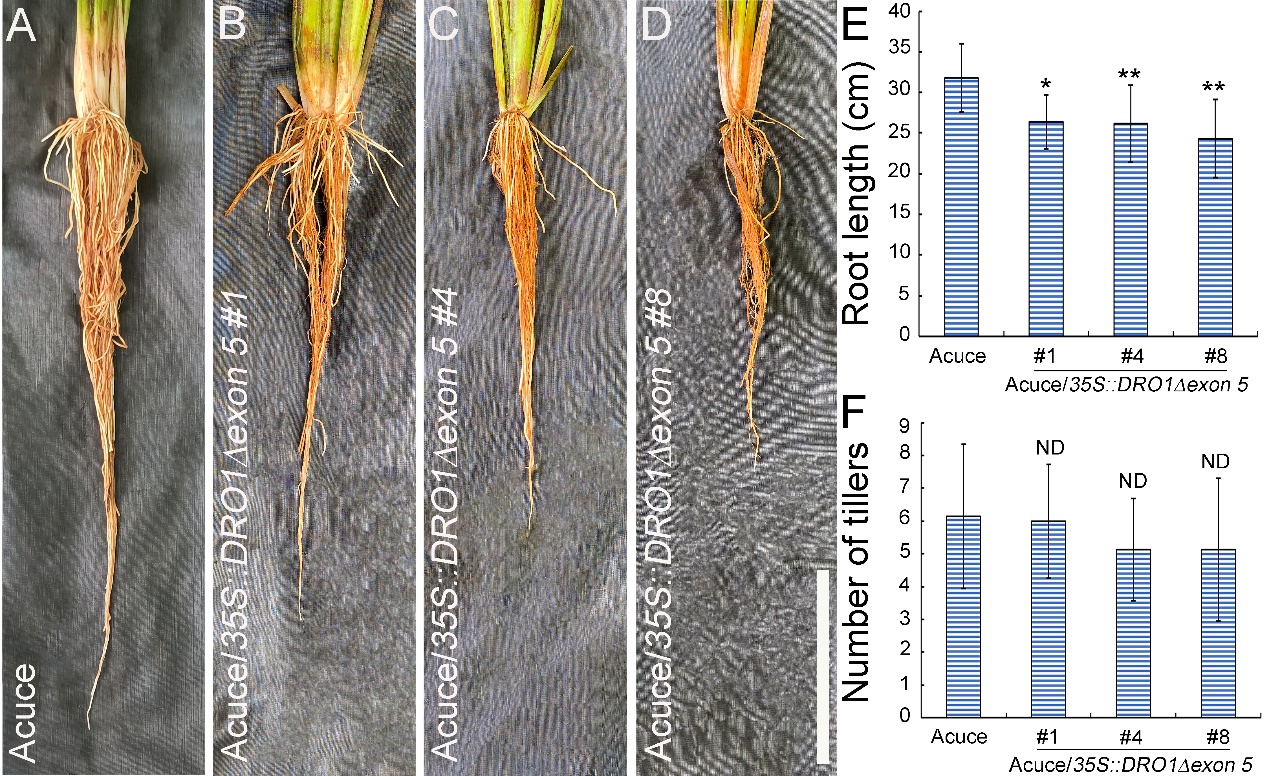


**Figure S7** Phenotypes of roots and tillers in transgenic Acuce rice lines

Roots and tillers of three-month-old wild-type Acuce (**A**) and transgenic Acuce rice lines (#1, #4, #8) grown in the experimental field with irrigation condition (**A-D**). Quantification of root lengths (**E**) (n_Acuce_ = 7, n_Acuce/_*_35S::DRO1Δexon5 #1_* = 7, n_Acuce/_*_35S::DRO1Δexon5 #4_* = 8, n_Acuce/_*_35S::DRO1Δexon5 #8_* = 7) and number of tillers (**F**) (n_Acuce_ = 7, n_Acuce/_*_35S::DRO1Δexon5 #1_* = 7, n_Acuce/_*_35S::DRO1Δexon5 #4_* = 8, n_Acuce/_*_35S::DRO1Δexon5 #8_* = 8) in wild-type Acuce and transgenic Acuce rice lines. Data are means ± SD. * *P* < 0.05, ** *P* < 0.01 (Student’s *t*-test). ND = no difference. Bar = 10 cm.


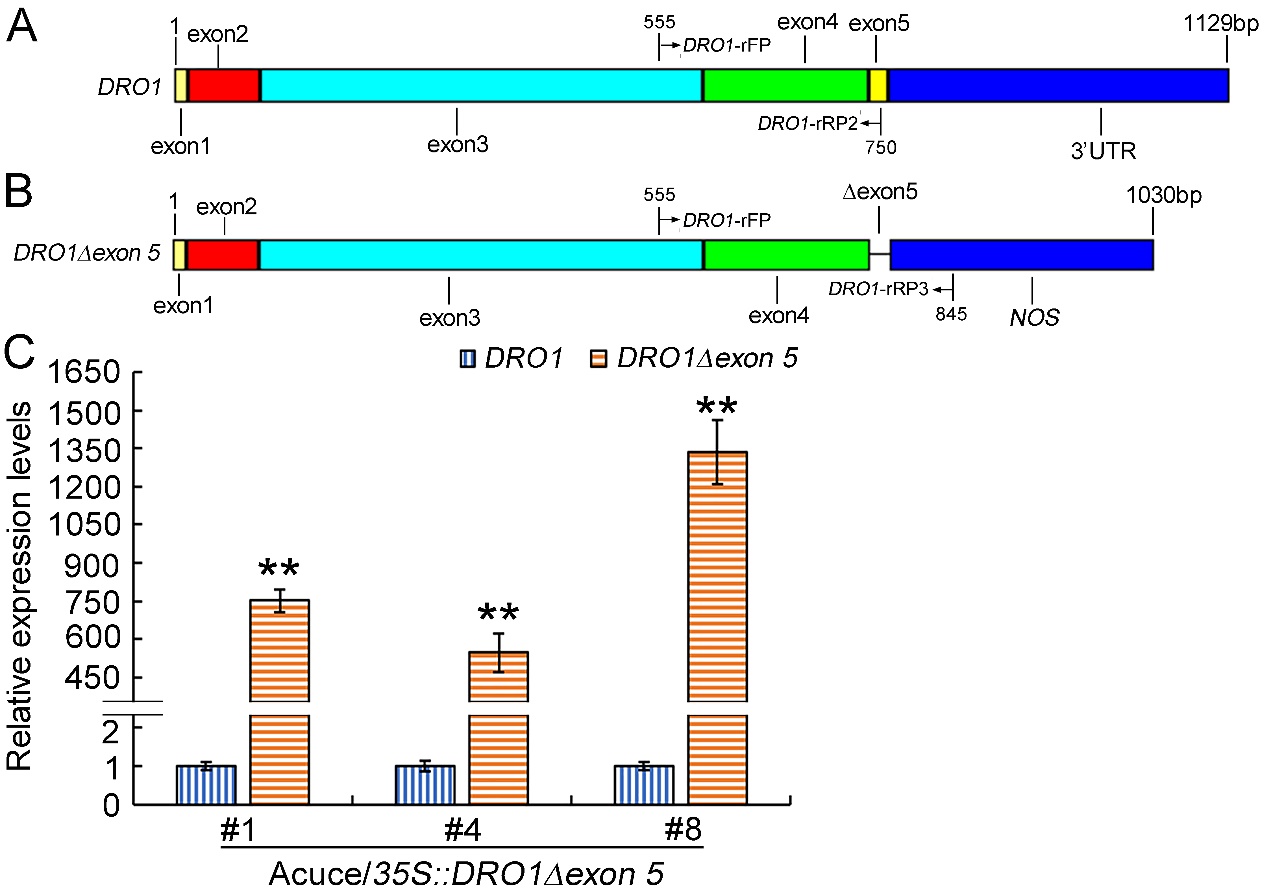


**Figure S8** Gene expression level of transgene *DRO1* in the transgenic Acuce rice lines.

Schematic diagram indicates the native full *DRO1* gene (**A**) and malfunctional *DRO1Δexon5* gene with a deletion of the exon 5 in the transgenic rice lines (#1, #4, #8) (**B**). The endogenous *DRO1* gene in transgenic lines #1, #4 and #8 was amplified with the primer pair DRO1-rFP and DRO1-rRP2 shown in the image (**A**), and the transgene *DRO1* in transgenic rice lines #1, #4 and #8 was amplified with the primer pair DRO1-rFP and DRO1-rRP3 shown in the image (**B**). The expression levels of endogenous and transgene *DRO1* in the primary root of tissue culture seedling of transgenic rice lines #1, #4 and #8 were detected by real-time PCR (**C**). The *Actin* gene was used as an internal control. The data presented here represent at least three biological replicates. Data are means ± SD; ***P* < 0.01 (SPSS analysis). The yellow, red, blue and green boxes indicate exons. Arrow points to the primers position. NOS = Nopaline synthase terminator. 3’ UTR = 3’ untranslated region.

**Table** **S1** Sequences of gene-specific primers.

| Target gene | Primer | Sequence of gene-specific primer | Locus |
| --- | --- | --- | --- |
| *ARF15* | ARF15-rFP | 5’-AATCATCATCTCCGCCATCTGT-3’ | Os05g0563400 |
|  | ARF15-rRP | 5’-GCCTATCATTCTCGTGCTTGTC-3’ |  |
| *CRL1* | CRL1-rFP | 5’-CTCCAGCAGCAGGTGATGAC-3’ | Os03g0149100 |
|  | CRL1-rRP | 5’-ACTGGTTGTAGCCGCCGTA-3’ |  |
| *DRO1* | DRO1-rFP | 5’-AATGGAGAAGTTGCTCAAGGCA-3’ | Os09g0439800 |
|  | DRO1-rRP1 | 5’-TCATCGTCTAGATCACGCAGTG-3’ |  |
|  | DRO1-rRP2 | 5’-TTCAAGCACA ATATACTCTGAATCA-3’ |  |
|  | DRO1-rRP3 | 5’-TGATAATCAT CGCAAGACCGG-3’ |  |
|  | DRO1-FP1 | 5’-CAGTGGTCTCACTCTATGAAGGTAAAGGCTCAAAC-3’ |  |
|  | DRO1-RP1 | 5’-CAGTGGTCTCACTGCCTACTTACACTCTGAATCAG-3’ |  |
|  | DRO1-FP2 | 5’-CTAGCAATCAAAGATCAAGC-3’ |  |
|  | DRO1-RP2 | 5’-CTTCTCCATTCTTGATTGAGG-3’ |  |
| *LEA19* | LEA19-rFP | 5’-AGGCGAGTGAGCAGGTGAA-3’ | Os05g0542500 |
|  | LEA19-rRP | 5’-TGGCAGAGGTGTCCTTGTTG-3’ |  |
| *LAZY1* | LAZY1-rFP | 5’-CGGCTACAAGACGATGAAGA-3’ | Os11g0490600 |
|  | LAZY1-rRP | 5’-GCTCTTCTTGGTCAGATTCC-3’ |  |
| *Os03g0624000* | Os03g0624000-rFP | 5’-TATTAGAAGGCAGGCAGCAG-3’ |  |
|  | Os03g0624000-rRP | 5’-TTGATGAGGTAGTGGTGAGTG-3’ |  |
| *Os06g0311000* | Os06g0311000-rFP | 5’-TCTTCGTTCTCATCGTCCTCAA-3’ |  |
|  | Os06g0311000-rRP | 5’-TTGTTGCTGCTGCCATCTTG-3’ |  |
| *Actin* | Actin-rFP | 5’-GAGTATGATGAGTCGGGTCCAG-3’ | Os11g0163100 |
|  | Actin-rRP | 5’-ACACCAACAATCCCAAACAGAG-3’ |  |
| *PIN1b* | PIN1b-rFP | 5’-ACATTGCCATTGTTCAGGCC-3’ | Os11g0137000 |
|  | PIN1b-rRP | 5’-AGCAGTATGTAGTAGACCAATG-3’ |  |
| *PIN1A* | PIN1A-rFP | 5’-CGTCTCGCTCGGACATCTA-3’ | Os02g0743400 |
|  | PIN1A-rRP | 5’-AGAAGTCGGT GTGGTTGAAG-3’ |  |
| *PIN1C* | PIN1C-rFP | 5’-ATCCTCATCACCGAGCAGTTC-3’ | Os06g0232300 |
|  | PIN1C-rRP | 5’-TATCTTGCCGTCCTCCTTCAC-3’ |  |
| *PIN2* | PIN2-rFP | 5’-ACATTTGCAATGGCAGTGAGGTTC-3’ | Os06g0660200 |
|  | PIN2-rRP | 5’-GGCAATTGTACTCCTTGGCAAACA-3’ |  |
| *qSOR1* | qSOR1-rFP | 5’-ACGATGCTTCACAAGAGGATGA-3’ | Os07g0614400 |
|  | qSOR1-rRP | 5’-CGGCTTATCCTCAAGATAGTAT-3’ |  |
| *SHR1* | SHR1-rFP | 5’-TACCACCACAGCAGCAACA-3’ | Os07g0586900 |
|  | SHR1-rRP | 5’-ACGACGACGAGGAGGAGAA-3’ |  |
| *TAC1* | TAC1-rFP | 5’-AACATCCAGGAACGACCAC-3’ | Os09g0529300 |
|  | TAC1-rRP | 5’-TAGGCTTTGAAGTGGATGCC-3’ |  |
| *WAXY* | WAXY-rFP | 5’-TACGGACCTGACACTGGAGTT-3’ | Os06g0133000 |
|  | WAXY-rRP | 5’-ACGAACACAACATCCTCACCAT-3’ |  |
| *WOX11* | WOX11-rFP | 5’-GCTCTACTACTCGTGTCAACCT-3’ | Os07g0684900 |
|  | WOX11-rRP | 5’-TGGAGGAGGATGCCGTACT-3’ |  |

| **Table S2** Root and tiller phenotypes in rice varieties of Yuanyang Hani’s terraced fields. | | | | | |  |
| --- | --- | --- | --- | --- | --- | --- |
| Varieties^a^ | Range of root length to width ratio | Length (cm) | Width (cm) | Tiller number | Root total  area (cm^2^) | |
| Qxg | 1.3-1.4 | 26.67 ± 7.86 | 19.88 ± 3.76 | 14.00 ± 6.16 | 113.53 ± 79.31 | |
| Dls |  | 29.14 ± 3.53 | 21.93 ± 1.71 | 19.00 ± 3.87 | 182.90 ± 24.26 | |
| Ljhj |  | 29.07 ± 0.69 | 21.55 ± 0.36 | 13.50 ± 0.71 | 196.05 ± 52.45 | |
| Mwg |  | 29.05 ± 3.67 | 22.29 ± 2.75 | 26.25 ± 4.03 | 206.50 ± 60.40 | |
| Ybg | 1.4-1.5 | 31.12 ± 6.15 | 21.57 ± 1.11 | 20.00 ± 6.12 | 232.76 ± 92.72 | |
| Mchn |  | 29.60 ± 4.36 | 20.29 ± 1.71 | 12.60 ± 4.22 | 163.90 ± 65.15 | |
| Heig |  | 29.48 ± 7.44 | 19.80 ± 4.32 | 18.60 ± 5.73 | 150.25 ± 59.89 | |
| Dd | 1.5-1.6 | 29.89 ± 7.34 | 19.70 ± 4.66 | 28.00 ± 7.21 | 147.17 ± 98.02 | |
| Ng |  | 32.76 ± 9.22 | 21.38 ± 3.70 | 19.00 ± 5.66 | 195.53 ± 87.37 | |
| Mlag |  | 30.33 ± 3.88 | 19.98 ± 2.87 | 20.00 ± 5.43 | 186.71 ± 66.54 | |
| Cb |  | 31.20 ± 3.67 | 20.59 ± 1.49 | 16.00 ± 3.61 | 192.21 ± 58.07 | |
| Huag |  | 35.80 ± 3.95 | 23.29 ± 1.87 | 28.33 ± 7.02 | 295.30 ± 73.86 | |
| Jyn |  | 34.59 ± 4.81 | 22.49 ± 1.93 | 17.20 ± 6.42 | 204.00 ± 47.49 | |
| Mzn |  | 30.16 ± 2.97 | 19.48 ± 1.61 | 15.33 ± 5.32 | 201.27 ± 83.49 | |
| Azg |  | 31.38 ± 7.34 | 19.92 ± 2.39 | 27.00 ± 4.90 | 188.06 ± 74.37 | |
| Cwn |  | 32.94 ± 1.38 | 21.11 ± 1.97 | 10.00 ± 2.65 | 199.64 ± 59.64 | |
| Yx |  | 28.87 ± 4.61 | 18.51 ± 1.45 | 14.00 ± 3.03 | 130.61 ± 37.20 | |
| Mlig |  | 37.60 ± 1.55 | 23.96 ± 1.51 | 32.50 ± 6.26 | 271.39 ± 55.90 | |
| Cz | 1.6-1.7 | 31.62 ± 4.74 | 19.57 ± 1.61 | 19.50 ± 7.05 | 163.24 ± 69.63 | |
| Gnhg |  | 33.11 ± 4.52 | 20.62 ± 1.44 | 16.75 ± 4.27 | 221.31 ± 65.79 | |
| Xg |  | 33.22 ± 2.41 | 20.66 ± 2.22 | 40.50 ± 6.36 | 276.04 ± 71.30 | |
| Cbg |  | 38.75 ± 8.09 | 23.49 ± 2.77 | 19.50 ± 9.19 | 331.39 ± 95.35 | |
| Sybg |  | 32.56 ± 4.47 | 19.64 ± 0.82 | 21.60 ± 4.16 | 189.17 ± 22.35 | |
| Gdg |  | 34.01 ± 5.51 | 20.61 ± 3.13 | 19.80 ± 6.98 | 191.00 ± 72.93 | |
| Bjg |  | 34.90 ± 6.45 | 20.97 ± 2.10 | 25.33 ± 5.51 | 221.02 ± 75.62 | |
| Mln | 1.7-1.8 | 39.68 ± 7.87 | 23.25 ± 3.40 | 19.00 ± 1.41 | 303.31 ± 53.50 | |
| Xbg |  | 32.20 ± 1.69 | 19.35 ± 3.03 | 13.33 ± 0.58 | 168.59 ± 41.95 | |
| Xpg |  | 34.56 ± 6.61 | 20.37 ± 1.96 | 17.00 ± 3.16 | 247.36 ± 44.41 | |
| Xhg |  | 28.86 ± 5.77 | 16.88 ± 1.35 | 13.00 ± 4.97 | 120.29 ± 54.56 | |
| Smc |  | 33.45 ± 5.07 | 19.87 ± 2.71 | 14.00 ± 4.85 | 162.40 ± 82.06 | |
| Hjlj |  | 33.63 ± 2.64 | 19.25 ± 1.25 | 14.25 ± 1.71 | 170.65 ± 50.43 | |
| Mxg |  | 40.08 ± 4.36 | 22.70 ± 3.58 | 27.25 ± 4.57 | 224.74 ± 39.73 | |
| Hkn | 1.8-1.9 | 36.68 ± 3.90 | 20.18 ± 2.75 | 12.80 ± 1.92 | 198.81 ± 56.75 | |
| Gtn |  | 33.87 ± 6.08 | 18.40 ± 1.39 | 20.25 ± 1.71 | 218.54 ± 14.34 | |
| Lccm |  | 36.62 ± 8.21 | 19.84 ± 1.96 | 18.60 ± 5.37 | 179.15 ± 66.03 | |
| Dpg |  | 38.14 ± 2.73 | 20.41 ± 1.39 | 18.40 ± 4.77 | 256.64 ± 28.78 | |
| Xhn |  | 33.70 ± 3.62 | 18.45 ± 4.22 | 21.20 ± 5.07 | 186.32 ± 61.39 | |
| Ajg |  | 31.98 ± 3.89 | 17.37 ± 2.63 | 16.80 ± 6.83 | 174.03 ± 63.17 | |

^a^ Root and tiller phenotypes of five-month-old rice seedlings grown in the experimental field with irrigation condition. Qi Xian Gu (Qxg), Da Leng Shui (Dls), Lao Jing Hong Jiao (Ljhj), Ma Wei Gu (Mwg), Ye Bai Gu (Ybg), Man Che Hong Nuo (Mchn), Hei Gu (Heig), Duo Dian (Dd), Nuo Gu(Ng), Meng La Gu (Mlag), Che Bu (Cb), Hua Gu (Huag), Jiu Yue Nuo (Jyn), Ma Zha Nuo (Mzn), Ai Zhe Gu (Azg), Chang Wei Nuo (Cwn), Yun Xiang (Yx), Mao Lai Gu (Mlig), Che Zuo (Cz), Ga Niang Hong Gu (Gnhg), Xiao Gu (Xg), Chuan Bai Gu (Cbg), Shi Yue Bai Gu (Sybg), Gan Di Gu (Gdg), Ban Jiu Gu (Bjg), Meng La Nuo (Mln), Xi Bai Gu (Xbg), Xiao Pi Gu (Xpg), Xiao Hua Gu (Xhg), Si Ma Che (Smc), Hong Jiao Lao Jing (Hjlj), Ma Xian Gu (Mxg), Hua Ke Nuo (Hkn), Gan Tian Nuo (Gtn), Le Che Che Ma (Lccm), Da Pi Gu (Dpg), Xiao Hua Nuo (Xhn), Ai Jiao Gu (Ajg).

| **Table S3** Details between the miRNAs and its target genes**.** | | | | |
| --- | --- | --- | --- | --- |
| **miRNA accession no.** | **Target accession no.** | **Target Name.** | **miRNA aligned fragment** | **Target aligned fragment** |
| Osa-miR156a | LOC_Os06g44970.1 | OsPIN2 | UGACAGAAGAGAGUGAGCAC | UAGCUCGUUCUCUUGUGUCA |
| Osa-miR156a | LOC_Os09g26840.1 | DRO1 | UGACAGAAGAGAGUGAGCAC | GUGUCUACUCUCUUCUGCCC |
| Osa-miR156b | LOC_Os06g44970.1 | OsPIN2 | UGACAGAAGAGAGUGAGCAC | UAGCUCGUUCUCUUGUGUCA |
| Osa-miR156b | LOC_Os09g26840.1 | DRO1 | UGACAGAAGAGAGUGAGCAC | GUGUCUACUCUCUUCUGCCC |
| Osa-miR156c | LOC_Os06g44970.1 | OsPIN2 | UGACAGAAGAGAGUGAGCAC | UAGCUCGUUCUCUUGUGUCA |
| Osa-miR156c | LOC_Os09g26840.1 | DRO1 | UGACAGAAGAGAGUGAGCAC | GUGUCUACUCUCUUCUGCCC |
| Osa-miR156e | LOC_Os06g44970.1 | OsPIN2 | UGACAGAAGAGAGUGAGCAC | UAGCUCGUUCUCUUGUGUCA |
| Osa-miR156e | LOC_Os09g26840.1 | DRO1 | UGACAGAAGAGAGUGAGCAC | GUGUCUACUCUCUUCUGCCC |
| Osa-miR156g | LOC_Os06g44970.1 | OsPIN2 | UGACAGAAGAGAGUGAGCAC | UAGCUCGUUCUCUUGUGUCA |
| Osa-miR156g | LOC_Os09g26840.1 | DRO1 | UGACAGAAGAGAGUGAGCAC | GUGUCUACUCUCUUCUGCCC |
| Osa-miR156i | LOC_Os06g44970.1 | OsPIN2 | UGACAGAAGAGAGUGAGCAC | UAGCUCGUUCUCUUGUGUCA |
| Osa-miR156i | LOC_Os09g26840.1 | DRO1 | UGACAGAAGAGAGUGAGCAC | GUGUCUACUCUCUUCUGCCC |
| Osa-miR156l | LOC_Os09g26840.1 | DRO1 | CGACAGAAGAGAGUGAGCAUA | UGUGUCUACUCUCUUCUGCCC |
| Osa-miR1846a | LOC_Os11g29840.1 | LAZY1 | AGUGAGGAGGCCGGGGCCGCUGGA | UUCGGCGGCAGCGGCCUUCUCACC |
| Osa-miR1846c | LOC_Os11g29840.1 | LAZY1 | AGUGAGGAGGCCGGGGCCGCU | GGCGGCAGCGGCCUUCUCACC |
| Osa-miR1875 | LOC_Os09g35980.1 | TAC1 | ACAAUGGAGUGAAGUGCAACAGAA | AUUUAUUACACUUUGUUCCAUGGU |
| Osa-miR5491 | LOC_Os07g42290.1 | qSOR1 | UGAAAUGGAGGCUCGUUGUAC | CAGCAACAAGUUUUCAUUUCA |
| Osa-miR5837 | LOC_Os09g35980.1 | TAC1 | GGUGAUGUGGAGCGUUCGGCA | UGCUUAACGCUCCACAUUAUC |
| Osa-miR5837 | LOC_Os09g35980.2 | TAC1 | GGUGAUGUGGAGCGUUCGGCA | UGCUUAACGCUCCACAUUAUC |
| Osa-miRN2268a | LOC_Os10g05690.1 | OsAUX4 | CCGCCGACGCCGACAGGAGCU | CGCUGCCGUCGGCGUCGGCGG |
| Osa-miRN2268a | LOC_Os11g29840.1 | LAZY1 | CCGCCGACGCCGACAGGAGCU | UGCUGCUGCCGGCGUCGGCGG |
| Osa-miRN2268a | LOC_Os11g29840.1 | LAZY1 | CCGCCGACGCCGACAGGAGCU | GGCUCCUG-CGGCGGCGGCGG |
| Osa-miRN2268b | LOC_Os10g05690.1 | OsAUX4 | CCGCCGACGCCGACAGGAGCU | CGCUGCCGUCGGCGUCGGCGG |
| Osa-miRN2268b | LOC_Os11g29840.1 | LAZY1 | CCGCCGACGCCGACAGGAGCU | UGCUGCUGCCGGCGUCGGCGG |
| Osa-miRN2268b | LOC_Os11g29840.1 | LAZY1 | CCGCCGACGCCGACAGGAGCU | GGCUCCUG-CGGCGGCGGCGG |
